# Mushroom-Derived Phenolic Compounds as Emerging Prebiotic-like Modulators of Gut Microbiota, Intestinal Health, and Metabolism

**DOI:** 10.3390/ph19071014

**Published:** 2026-06-30

**Authors:** Juliana Garcia, Eva Olo-Fontinha, Jani Silva, Rui Dias-Costa, Maria José Alves, Irene Gouvinhas

**Affiliations:** 1 AquaValor—Centro de Valorização e Transferência de Tecnologia da Água—Associação, Rua Dr. Júlio Martins n.º 1, 5400-342 Chaves, Portugal; evafontinha@hotmail.com (E.O.-F.); maria.alves@ipb.pt (M.J.A.); 2 LiveWell—Research Centre for Active Living & Wellbeing, Instituto Politécnico de Bragança, 5300-253 Bragança, Portugal; 3 Centre for the Research and Technology of Agroenvironmental and Biological Sciences, CITAB, Inov4Agro, Universidade de Trás-os-Montes e Alto Douro, UTAD, Quinta de Prados, 5000-801 Vila Real, Portugal; ruiacosta@utad.pt; 4 Laboratório Associado, Escola Superior de Biotecnologia, CBQF—Centro de Biotecnologia e Química Fina, Universidade Católica Portuguesa, Rua Diogo Botelho 1327, 4169-005 Porto, Portugal; 5 CIMO—Centro de Investigação de Montanha, Instituto Politécnico de Bragança, 5300-253 Bragança, Portugal; 6 Animal and Veterinary Research Center (CECAV), University of Trás-os-Montes and Alto Douro, 5000-801 Vila Real, Portugal; 7 Associate Laboratory of Animal and Veterinary Sciences (AL4AnimalS), 1300-477 Lisboa, Portugal

**Keywords:** mushroom phenolics, phenolic acids, gut microbiota, prebiotic-like activity, colonic fermentation, microbial metabolites, intestinal barrier, short-chain fatty acids, metabolic health, functional foods

## Abstract

**Background/Objectives:** Mushroom-derived phenolic compounds are gaining attention as bioactive molecules with potential roles in gut microbiota modulation, intestinal health, and metabolic regulation. Although mushroom polysaccharides are well established as fermentable substrates, the contribution of fungal phenolics to microbiota–host interactions remains less defined. This review aimed to critically analyse the evidence supporting mushroom-derived phenolic compounds as emerging prebiotic-like modulators of gut microbiota, intestinal function, and host metabolism. **Methods:** A narrative critical review was conducted using scientific literature retrieved from PubMed, Scopus, Web of Science, and Google Scholar. Studies addressing phenolic profiling in edible and medicinal mushrooms, gastrointestinal digestion, colonic fermentation, microbial biotransformation, gut microbiota modulation, intestinal barrier function, inflammation, and metabolic outcomes were considered. Particular attention was given to chromatographic and mass spectrometry-based studies, in vitro digestion/fermentation models, mechanistic studies, animal experiments, clinical trials, systematic reviews, and meta-analyses. **Results:** Current evidence shows that mushrooms contain diverse phenolic compounds, mainly phenolic acids such as gallic, protocatechuic, caffeic, *p*-coumaric, ferulic, vanillic, syringic, and cinnamic acids. Due to limited small intestine absorption, a substantial fraction of these compounds may reach the colon, where they undergo microbial biotransformation into smaller phenolic metabolites. These metabolites may influence microbial ecology, support beneficial taxa, modulate short-chain fatty acid production indirectly, attenuate oxidative stress and inflammatory signaling, and contribute to intestinal barrier integrity. However, most evidence derives from in vitro and preclinical studies, while human data remain limited and are mainly based on whole-mushroom interventions. **Conclusions:** Mushroom-derived phenolic compounds are promising prebiotic-like modulators within the microbiota–metabolite–host axis. Nevertheless, their specific contribution cannot yet be quantitatively distinguished from that of other mushroom constituents, particularly β-glucans, chitin, and other fungal polysaccharides, because most available evidence derives from whole-mushroom matrices, crude extracts, or polysaccharide-rich preparations rather than isolated phenolic fractions. Future studies should compare whole mushroom preparations, polysaccharide-rich fractions, and standardized phenolic-rich extracts, integrating metabolomics, microbiome profiling, and well-designed clinical trials to clarify the relative mechanistic and therapeutic relevance of mushroom phenolics. Future studies should use standardized phenolic-rich extracts, metabolomics, microbiome analysis, and well-designed clinical trials to clarify their mechanistic relevance, clinical significance, and translational potential.

## 1. Introduction

Mushrooms are increasingly recognized as functional foods providing a complex matrix of bioactive compounds, including β-glucans, chitin, ergothioneine, terpenoids, sterols, vitamins, minerals, and phenolic compounds [1,2]. Although fungal polysaccharides have traditionally received most attention for their immunomodulatory and prebiotic potential, mushroom-derived phenolic compounds are emerging as relevant modulators of oxidative stress, inflammation, gut microbial ecology, and host metabolism [3,4]. This interest is particularly timely because intestinal dysbiosis, impaired epithelial barrier function, chronic low-grade inflammation, and altered microbial metabolite production are now considered central mechanisms linking diet to metabolic disorders, including obesity, type 2 diabetes, and cardiometabolic disease [5,6].

Phenolic compounds are secondary metabolites characterized by one or more hydroxylated aromatic rings. In edible mushrooms, the most consistently reported phenolics are phenolic acids, including gallic, protocatechuic, *p*-hydroxybenzoic, vanillic, syringic, caffeic, *p*-coumaric, ferulic, cinnamic, and chlorogenic acids, although their abundance varies markedly according to species, strain, cultivation system, developmental stage, anatomical part, geographic origin, processing, and extraction method [3,7,8]. Recent LC-MS/MS and high-resolution mass spectrometry studies have shown that commonly consumed mushrooms such as *Agaricus bisporus*, *Lentinula edodes*, *Pleurotus ostreatus*, *Pleurotus eryngii*, *Pholiota nameko*, and *Flammulina velutipes* contain diverse phenolic profiles, with phenolic acids generally predominating over other phenolic subclasses [7,8]. However, the presence and biosynthetic origin of flavonoids in fungi remain controversial, as some compounds tentatively assigned as flavonoids may reflect analytical limitations, environmental inputs, substrate-derived compounds, or misannotation in untargeted metabolomic workflows [3,8].

The biological relevance of dietary phenolics is closely linked to their limited small-intestinal absorption. It has been estimated that only approximately 5–10% of ingested polyphenols are absorbed in the upper gastrointestinal tract, whereas 90–95% may reach the colon, where they become available for microbial transformation [9,10]. This low bioavailability should not be interpreted as a limitation alone, since the colon represents a metabolically active interface where phenolic compounds can interact bidirectionally with the gut microbiota. Microbial enzymes, including glycosidases, esterases, tannases, decarboxylases, reductases, and dioxygenases, convert complex phenolic structures into lower-molecular-weight metabolites that may display greater bioavailability and distinct biological activities compared with their parent compounds [10,11]. At the same time, phenolics and their metabolites may influence microbial composition and function by exerting selective antimicrobial pressure, supporting beneficial taxa, modulating redox conditions, and affecting cross-feeding interactions [11,12].

These properties have led to increasing discussion of phenolic compounds as “prebiotic-like” molecules. According to the International Scientific Association for Probiotics and Prebiotics, a prebiotic is “a substrate that is selectively utilized by host microorganisms conferring a health benefit” [13]. While some polyphenols may partially meet this concept, their classification as true prebiotics remains debated because they are not uniformly fermented as primary energy substrates and their effects are often compound-, dose-, matrix-, microbiota-, and host-dependent [12,13,14]. Therefore, the term “prebiotic-like” is more appropriate for mushroom-derived phenolics at present. It acknowledges their capacity to modulate microbial ecology and microbial metabolite production without assuming that they consistently fulfil the strict criteria of selective utilization and demonstrated causal health benefit required for classical prebiotics [13,14].

Evidence specifically addressing mushroom-derived phenolic compounds and the gut microbiota remains limited but promising. In vitro digestion and colonic fermentation studies indicate that phenolic-containing mushroom matrices can modulate beneficial bacterial groups, reduce potentially harmful taxa, increase lactic acid and short-chain fatty acid (SCFA) production, and lower colonic pH. For example, digested *Pleurotus djamor* powder promoted increases in *Lactobacillus* spp./*Enterococcus* spp., *Bifidobacterium* spp., and *Ruminococcus albus*/*R. flavefaciens*, while reducing *Clostridium histolyticum* during in vitro colonic fermentation [15]. These findings suggest that mushroom matrices may create a colonic environment favorable to saccharolytic fermentation and microbial metabolite production. However, because mushrooms contain multiple fermentable and bioactive components, including β-glucans, chitin, heteropolysaccharides, proteins, and phenolics, the specific contribution of phenolic compounds remains difficult to isolate.

From a health perspective, mushroom-derived phenolics may contribute to intestinal and metabolic homeostasis through complementary mechanisms. Locally, phenolic metabolites may reduce oxidative stress, attenuate inflammatory signaling pathways such as NF-κB and MAPK, support epithelial barrier integrity, and interact with microbial communities involved in SCFA production [5,11,12]. Systemically, absorbed phenolic metabolites may influence glucose and lipid metabolism by modulating oxidative stress, insulin signaling, mitochondrial function, inflammatory tone, bile acid metabolism, and gut hormone secretion [5,6,12]. Nevertheless, current human evidence remains stronger for whole-mushroom consumption than for isolated mushroom phenolics. Systematic reviews suggest that mushroom intake may improve selected cardiometabolic markers, particularly triglycerides and high-sensitivity C-reactive protein, but findings remain heterogeneous and are limited by differences in mushroom species, intervention design, dose, duration, and lack of detailed phytochemical, microbiome, and metabolomic characterization [16,17].

Thus, although mushroom-derived phenolic compounds are mechanistically promising, their role as prebiotic-like modulators of gut microbiota and metabolism remains insufficiently defined. Important gaps persist regarding their digestive stability, colonic bioaccessibility, microbial biotransformation, metabolite profiles, dose–response relationships, interindividual variability, and causal contribution to clinical outcomes. This review therefore aims to critically examine the current evidence on mushroom-derived phenolic compounds as emerging prebiotic-like modulators of gut microbiota, intestinal health, and host metabolism. Particular attention is given to their chemical diversity, limited intestinal absorption, colonic fate, microbial biotransformation, effects on gut barrier and inflammatory pathways, and potential relevance in chronic metabolic disorders. By integrating chemical, microbiological, mechanistic, and clinical evidence, this review highlights both the promise and the current limitations of mushroom phenolics as functional modulators within the microbiota–metabolite–host axis.

## 2. Materials and Methods

This article was designed as a critical narrative review, not as a systematic review or meta-analysis. The literature search was structured to improve transparency and thematic coverage, but it was not intended to provide an exhaustive, reproducible systematic evidence synthesis. Therefore, no PRISMA flow diagram, protocol registration, duplicate independent screening, formal risk-of-bias assessment, or pooled quantitative analysis was performed. Instead, studies were selected purposively according to their relevance to the chemical characterization, gastrointestinal fate, microbial biotransformation, microbiota modulation, intestinal effects, and metabolic implications of mushroom-derived phenolic compounds.

### 2.1. Literature Search Strategy

This critical narrative review was supported by a structured literature search designed to ensure broad and balanced coverage of the available evidence on mushroom-derived phenolic compounds, gut microbiota modulation, intestinal health and host metabolism. The search strategy was developed to identify relevant chemical, microbiological, mechanistic, preclinical and clinical studies, as well as key conceptual publications related to prebiotic terminology, polyphenol bioavailability and microbiota-dependent metabolism.

The bibliographic search was primarily performed using PubMed, Scopus and Web of Science. Google Scholar was used only as a complementary source to identify additional relevant publications, citation-linked studies and key conceptual papers that may not have been retrieved through the main bibliographic databases. Additional relevant studies were also identified through manual screening of reference lists from selected articles and key reviews.

The literature search primarily covered studies published between January 2010 and March 2026, although seminal and highly relevant earlier publications were also included when necessary to provide conceptual or mechanistic background.

The search strategy combined controlled vocabulary terms and free-text keywords related to mushrooms, phenolic compounds, gut microbiota, prebiotic-like activity, intestinal health, inflammation and metabolic disorders. Search terms were organized into three main conceptual blocks: mushroom-related terms, phenolic compound-related terms and gut microbiota/intestinal health-related terms. Representative Boolean combinations included: (“mushroom phenolics” OR “mushroom polyphenols” OR “edible mushrooms” OR “medicinal mushrooms”) AND (“phenolic acids” OR “phenolic compounds” OR “polyphenols”) AND (“gut microbiota” OR “gut microbiome” OR “intestinal health” OR “prebiotic-like” OR “microbial metabolism” OR “short-chain fatty acids” OR “SCFA” OR “intestinal barrier” OR “inflammation” OR “metabolic syndrome” OR “obesity” OR “type 2 diabetes” OR “cardiometabolic health” OR “colonic fermentation”). Boolean operators were adapted to the syntax of each database to optimize retrieval.

To control relevance and study quality, records identified through Google Scholar were not considered automatically eligible. They were included only when they corresponded to peer-reviewed scientific articles, systematic reviews, meta-analyses or highly relevant conceptual publications directly aligned with the scope of the review. Non-peer-reviewed materials, editorials, patents, conference abstracts, blog posts and documents lacking sufficient methodological detail were excluded.

### 2.2. Literature Inclusion Criteria

Studies were considered eligible when they fulfilled at least one of the following criteria: (i) characterization of phenolic compounds in edible or medicinal mushrooms; (ii) investigation of gastrointestinal digestion, intestinal bioaccessibility, colonic fermentation, or microbial biotransformation of mushroom-derived phenolics; (iii) evaluation of gut microbiota modulation associated with mushroom phenolics or phenolic-rich mushroom extracts; (iv) assessment of intestinal, metabolic, anti-inflammatory, antioxidant, or cardiometabolic effects associated with mushroom-derived phenolic compounds; or (v) mechanistic studies related to microbiota–metabolite–host interactions.

Both experimental and observational evidence were considered, including in vitro gastrointestinal digestion and fermentation models, cell culture studies, animal studies, clinical trials, systematic reviews, and meta-analyses. Particular emphasis was given to recent studies employing advanced analytical methodologies, including High-Performance Liquid Chromatography with Diode-Array Detection (HPLC-DAD), Liquid Chromatography coupled to Tandem Mass Spectrometry (LC-MS/MS), and Ultra-High-Performance Liquid Chromatography coupled to Quadrupole Time-of-Flight Tandem Mass Spectrometry (UHPLC-QTOF-MS/MS), and metabolomics-based approaches for phenolic profiling and microbial metabolite identification.

Studies were excluded when they: (i) did not specifically evaluate mushrooms or mushroom-derived phenolic compounds; (ii) focused exclusively on non-phenolic mushroom constituents without relevance to the scope of this review; (iii) lacked sufficient methodological detail; (iv) were conference abstracts, editorials, patents, or non-peer-reviewed documents; or (v) were not available in English.

### 2.3. Literature Selection and Information Synthesis

Publications were selected according to their thematic relevance, methodological robustness and contribution to the main domains of the review. Priority was given to studies addressing phenolic profiling in mushrooms, gastrointestinal digestion and bioaccessibility, colonic fermentation, microbial biotransformation, gut microbiota modulation, intestinal barrier function, inflammatory regulation, SCFA-related responses and cardiometabolic outcomes. Information from the selected studies was synthesized narratively, with particular attention to mushroom species, extraction method, analytical approach, major phenolic compounds detected, experimental model, microbiota-related outcomes, microbial metabolites and reported biological effects.

For studies related to gut microbiota modulation and metabolic health, additional information regarding microbial taxa, SCFA production, inflammatory markers, oxidative stress parameters, intestinal permeability markers, and metabolic outcomes was also collected whenever available.

### 2.4. Phenolic Compound Classification and Analytical Data Compilation

Phenolic compounds identified in mushrooms were classified according to their chemical subclasses, including hydroxybenzoic acids, hydroxycinnamic acids, flavonoids, stilbenes, lignans, and other phenolic-related compounds. Special emphasis was placed on phenolic acids due to their predominance in edible mushroom species.

Only studies using chromatographic or mass spectrometry-based analytical techniques capable of individual phenolic compound quantification were included in Table S1. These methodologies included LC-MS, LC-MS/MS, and Triple TOF-LC-MS/MS. Among the studies included, only Erbiai et al. [18] and Radzki et al. [7] performed quantitative analyses based on calibration curves established for each individual phenolic compound identified, allowing for more accurate and compound-specific quantification. In contrast, Calleja-Gómez et al. [8] relied on an external calibration approach using a representative compound for each phenolic group, rather than individual standards for each analyte.

### 2.5. Scope and Conceptual Approach

Given the heterogeneity of available evidence and the limited number of studies specifically evaluating isolated mushroom-derived phenolic compounds, this work was designed as a critical narrative review rather than a systematic review or meta-analysis. The aim was not to quantify the total number of eligible studies or generate pooled effect estimates, but to integrate evidence from chemistry, microbiology, nutrition, gastrointestinal physiology, and metabolic health in order to critically discuss the emerging concept of mushroom-derived phenolic compounds as prebiotic-like modulators within the microbiota–metabolite–host axis.

Study selection followed a relevance-based filtering process. After the initial database searches and manual reference screening, titles and abstracts were reviewed to exclude studies clearly outside the scope of the review. Full texts were then assessed for thematic relevance, methodological adequacy, and contribution to at least one of the main review domains: phenolic profiling, gastrointestinal digestion, colonic bioaccessibility, microbial biotransformation, gut microbiota modulation, intestinal barrier function, inflammatory regulation, SCFA-related responses, and cardiometabolic outcomes. Priority was given to original experimental studies, clinical studies, systematic reviews, meta-analyses, and mechanistic papers using robust analytical or microbiological approaches. Seminal publications were included when necessary to support conceptual definitions, particularly regarding prebiotic terminology, polyphenol bioavailability, and microbiota-dependent metabolism.

Particular attention was given to distinguishing direct effects of mushroom phenolic compounds from effects potentially mediated by other mushroom constituents, including β-glucans, chitin, heteropolysaccharides, dietary fiber, proteins, terpenoids, and associated fungal metabolites. Because of the narrative design, study selection numbers and exclusion counts were not tabulated; however, the selection and filtering criteria are now explicitly described to clarify the scope and reproducibility limits of the review.

## 3. Phenolic Compounds and Their Interaction with the Gut Microbiota

### 3.1. Major Phenolic Compounds Classes in Mushrooms

Mushrooms contain a wide range of phenolic compounds, which contribute to their antioxidant, antimicrobial, and other health-related activities. Studies have shown that these biological effects are largely associated with their high levels of polysaccharides, although phenolic compounds have shown promising properties [19].

Although fungi share certain biosynthetic pathways with plants for the production of phenolic compounds, such as melanin precursors, the ability of mushrooms to synthesize flavonoids remains highly debated [20]. The present review compiles and discusses studies published within the last five-year period, from 2021 onwards, which are presented in Table S1.

Overall, the phenolic profile of edible mushrooms varied considerably across species, mushroom tissues, and extraction approaches. Consistent with this conceptual framework, the critical analysis of the studies compiled in Table S1 confirms that phenolic acids represent the most consistently reported class of phenolic compounds in edible mushrooms analyzed using HPLC-based techniques. Cinnamic acid emerged as the most abundant compound, particularly in wild mushrooms such as *Armillaria mellea* and *Macrolepiota procera*, where concentrations exceeded 80 µg/g dry weight (DW). In contrast, cultivated mushrooms generally showed much lower levels of this compound when aqueous or ethanolic extraction was applied.

Other phenolic compounds, including 4-hydroxybenzaldehyde, caffeic acid and ferulic acid, were typically detected at low concentrations, often below 1 µg/g DW, especially in cultivated species. Protocatechuic acid and syringic acid displayed intermediate levels, with clear differences not only among species but also between caps, stems and whole fruiting bodies. Moreover, the extraction method played a relevant role in phenolic recovery, as pulsed electric fields (PEF)-assisted aqueous extraction consistently increased the levels of selected compounds, such as 3,5-dicaffeoylquinic acid and cinnamic acid, compared to conventional extraction. Taken together, these results underline the combined influence of mushroom species, tissue differentiation, geographical origin, and extraction strategy on the phenolic composition of edible mushrooms [4,7,18].

Phenolic compounds were unevenly distributed within mushroom fruiting bodies, with caps generally showing higher concentrations than stems, while whole fruiting bodies presented intermediate values. This trend was consistent for several phenolic acids, although exceptions were observed depending on the compound and species, highlighting tissue-specific accumulation patterns.

**Figure 1:**
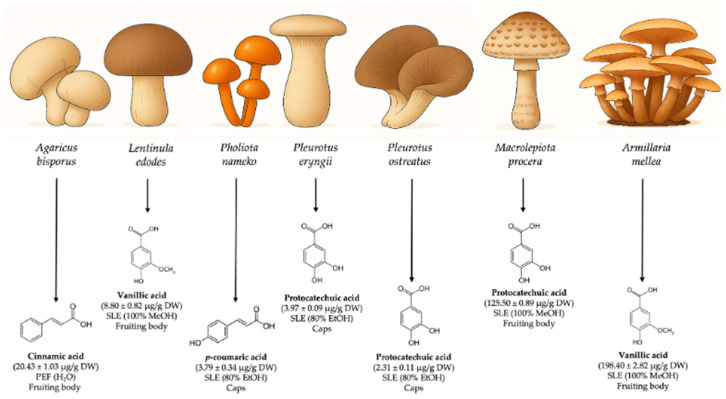
Summary of edible mushroom species and their most highly quantified phenolic acids, based on the maximum concentrations reported in HPLC-based studies compiled in Table S1. DW, dry weight; SLE, solid liquid extraction; PEF, pulsed electric fields.

Figure 1 summarizes the edible mushroom species included in Table S1 together with their most highly quantified phenolic acid. For each species, the compound shown corresponds to the highest concentration reported across HPLC-based studies, providing a simplified overview of the dominant phenolic compound.

Chu et al. used LC-ESI-QTOF-MS/MS to tentatively identify a wide range of phenolic compounds in different edible mushroom species, including shiitake (*Lentinula edodes*), enoki (*Flammulina velutipes*), white button (*Agaricus bisporus*), Swiss brown (*Agaricus bisporus*), white flat (*Agaricus bisporus*), portobello flat (*Agaricus bisporus*), organic white (*Agaricus bisporus*), white cup (*Agaricus bisporus*), and oyster (*Pleurotus ostreatus*). However, compound identification was based primarily on the molecular ions (theoretical and observed *m*/*z* values) and retention times, without detailed MS/MS fragmentation data. Phenolic acids were mainly represented by cinnamic acid, with a retention time of 4.53 min and detected in negative ion mode ([M−H]^−^, *m*/*z* = 147.0463), which was detected in most of the analyzed mushroom samples. In contrast, *p*-coumaroyl malic acid, with a retention time of 3.65 min was primarily identified in white flat, organic white, portobello flat, and shiitake mushrooms, constituting a novel finding in fungal matrices [4]. In addition, several flavonoid subclasses, including isoflavonoids, flavonols, flavanones, flavanols, dihydroflavonols, and anthocyanins, were tentatively characterized with species-dependent distribution patterns. However, the identification of flavonoids in fungal matrices should be interpreted with caution, as their occurrence in mushrooms remains a matter of ongoing debate. For instance, a recent study has reported the presence of flavonoids in certain species despite the apparent absence of key genes involved in their canonical biosynthetic pathways, raising concerns about their origin and the potential for misannotation [21]. These limitations are particularly relevant when identification is based solely on accurate mass data without comprehensive MS/MS confirmation or validation with authentic standards. Lignans, stilbenes, and other phenolic compounds were also detected in selected mushroom species, further highlighting the chemical diversity of phenolic compounds revealed by high-resolution mass spectrometry.

### 3.2. Limited Intestinal Absorption of Phenolic Compounds

The absorption of phenolic compounds in the gastrointestinal tract is naturally limited by several structural, chemical, and physiological factors. First, the food matrix and fibre content play a determining role. Many phenolics are tightly bound to indigestible cell walls and dietary fibre, which hinders their release during digestion. As a consequence, absorption in the small intestine is delayed or even prevented, causing a significant portion of the ingested dose to reach the colon intact [22]. Beyond the influence of the food matrix, the chemical form and molecular size of phenolics strongly influence their bioavailability. These compounds are often present in the form of glycosides, esters, polymers, or tannins, which cannot be directly absorbed by the intestinal mucosa. Therefore, for absorption to occur, prior hydrolysis is necessary, often dependent on the intestinal microbiota. In this context, high molecular weight forms, such as proanthocyanidins, have particularly low absorption rates due to their structural complexity [23,24]. However, even when phenolic compounds are successfully absorbed, the amount of phenolic compound that reaches the systemic circulation is reduced due to transport and efflux mechanisms, as well as extensive metabolism. Transporters such as P-glycoprotein can return absorbed compounds to the intestinal lumen, further limiting their availability. In parallel, extensive phase I and II metabolism occurs in enterocytes and the liver, leading to a rapid transformation of parent compounds into metabolites, significantly reducing the concentration of these compounds in circulation [24]. Another important gut-level barrier is competition among phenolic compounds, whereby the presence of one phenolic may reduce the intestinal absorption of another [22]. Silberberg et al. [25] reported a competitive interaction between quercetin and catechin during digestion, resulting in a decreased intestinal absorption of quercetin and a potential delay in catechin absorption over time. Together, these factors explain why the absorption of phenolics is limited and why their biological action depends not only on ingestion but also on their transformation and interaction along the gastrointestinal tract [26,27]. As a result, a substantial fraction of ingested phenolic compounds escapes absorption in the small intestine and reaches the colon largely intact, where they become available for microbial metabolism and may contribute to local microbiota-related effects, particularly within complex food matrices [25,26]. Only about 5–10% phenolic compounds are absorbed in the small intestine, with the majority (90–95%) passing unmetabolized to the large intestine [28], and becomes available in the colonic lumen [11,24].

### 3.3. Colonic Availability and Microbial Biotransformation of Phenolic Compounds

Once in the colon, phenolic compounds display increased availability for interaction with the gut environment, particularly within the highly dense microbial ecosystem of the large intestine [29]. At this stage, *Clostridium* and *Eubacterium* are recurrent genera involved in phenolic metabolism, but many responsible strains and pathways remain unidentified [30]. However, evidence from dietary polyphenol metabolism, mostly derived from plant-based matrices, indicates that phenolic biotransformation is not restricted to these genera. Other anaerobic or facultative anaerobic taxa, including members of *Eggerthella*, *Gordonibacter*, *Adlercreutzia*, *Slackia*, *Bacteroides*, *Bifidobacterium*, *Lactobacillus*/*Lacticaseibacillus*, and *Enterococcus*, have been implicated in different steps of polyphenol conversion, including hydrolysis, deconjugation, reduction, dehydroxylation, demethylation, decarboxylation, and aromatic ring cleavage. Although these taxa have been more extensively characterized in the context of plant polyphenols, they provide a useful mechanistic framework for interpreting the potential microbial transformation of mushroom-derived phenolic acids. *Enterococcus casseliflavus* has been reported to hydrolyse and ferment the glycosidic moiety of quercetin-*O*-glycosides, releasing the quercetin aglycone, although it is unable to further catabolise this compound. In contrast, *Eubacterium ramulus* is involved in the subsequent degradation of the aglycone, generating lower-molecular-weight phenolic metabolites [31]. Ellagitannins and ellagic acid are metabolised by bacterial genera such as *Gordonibacter*, *Ellagibacter*, and *Enterocloster*, leading to the formation of urolithins [32]. Other genera implicated in colonic phenolic biotransformation include *Bifidobacterium*, *Lactobacillus*, *Streptococcus*, *Ruminococcus*, *Eggerthella*, *Peptostreptococcus*, *Escherichia* [33]. In in vitro fermentation studies using phenolic-rich substrates, several gut microbial taxa have been associated with the production of phenolic metabolites. For instance, members of the genera *Faecalibacterium*, *Oscillibacter*, and *Dialister*, as well as bacteria belonging to the *Ruminococcaceae* family, have shown positive correlations with the generation of microbial-derived phenolic metabolites following the fermentation of *Psidium guajava* fractions. Similarly, the fermentation of phenolic compounds from yarrow and marjoram has been associated with increased abundances of *Bifidobacterium*, *Collinsella*, *Romboutsia*, *Akkermansia*, *Blautia*, and *Dialister*. These microbial shifts occurred concomitantly with the formation of bioactive metabolites, including phloroglucinol and various hydroxyphenylpropionic and hydroxybenzoic acids, highlighting the important role of the gut microbiota in the biotransformation of dietary phenolics into potentially bioavailable compounds. Importantly, the extent of these microbial biotransformations depends not only on microbial composition but also on factors such as food matrix, processing, fibre binding, and host factors all of which influence how much phenolic material reaches the colon and what metabolites are ultimately produced [28]. In particular, phenolic compounds bound to fibre-rich matrices are especially prone to liberation under colonic conditions, where they become accessible to microbial enzymes [34,35]. This mechanism is especially relevant for mushrooms, because phenolic acids may be physically entrapped within or associated with β-glucans, chitin, mannans, proteins, and other fungal cell-wall components. Under colonic conditions, microbial degradation of these structural polysaccharides may facilitate the release of bound or matrix-associated phenolics, thereby increasing their local availability for further microbial metabolism. Consistent with this, evidence from in vitro colonic fermentation models consistently shows a marked reduction in measurable phenolic content during the colonic phase, reflecting extensive microbial utilization and metabolic processing [24,36,37]. For example, according to Cañas et al. [36,37], phenolic acids exhibited the highest bioaccessibility in the colonic phase, whereas flavonols and flavones were degraded during gastrointestinal digestion.

In the colonic environment, resident microorganisms extensively biotransform phenolic compounds (through deglycosylation, ring fission, dihydroxylation, decarboxylation and oxidation-reduction reactions) into a wide range of lower–molecular–weight metabolites, including phenolic acids, tyrosols, lignans, and other small phenolics, which are often more bioavailable than their parent compounds [38]. This metabolic capacity is supported by a diverse range of enzymatic activities, including hydrolytic enzymes (such as tannases and glycosidases, e.g., α-L-rhamnosidase and β-glucosidase), as well as esterases, phenolic acid reductases, decarboxylases, dehydroxylases, demethylases, and dioxygenases [30,39,40]. More specifically, β-glucosidases and α-L-rhamnosidases participate in the cleavage of glycosidic bonds and the release of aglycones; esterases, feruloyl esterases, and tannases contribute to the liberation of phenolic acids esterified to fibre, proteins, or other matrix components; phenolic acid decarboxylases may convert hydroxycinnamic acids such as caffeic, ferulic, and p-coumaric acids into vinyl derivatives; and vinylphenol reductases and hydroxycinnamate reductases may further reduce these intermediates into ethylphenols or hydroxyphenylpropionic acid derivatives. In addition, demethylases, dehydroxylases, and dioxygenases can contribute to the structural simplification of aromatic compounds, leading to smaller metabolites such as hydroxyphenylacetic, hydroxyphenylpropionic, benzoic, vanillic, homovanillic, and hippuric acid derivatives. As a consequence, these microbial-derived metabolites tend to accumulate in the colonic lumen and may subsequently be absorbed into the systemic circulation, frequently exhibiting bioactivities that differ from those of the original phenolic structures [41]. Beyond systemic absorption, non-absorbed phenolic compounds and their microbial metabolites play an important role at the local intestinal level. Through their continuous presence in the colon, these compounds can modulate gut microbial composition and metabolic activity, influencing colonic metabolism, including the production of short-chain fatty acids (acetate, propionate, and butyrate), the regulation of inflammatory responses [38], and suppressing opportunistic bacteria [42]. Nevertheless, SCFAs should not be interpreted as direct products of phenolic metabolism. Rather, phenolic compounds may indirectly influence SCFA production by reshaping microbial ecology, modulating antimicrobial pressure, altering redox conditions, and affecting cross-feeding interactions among saccharolytic, lactate-producing, and butyrate-producing bacteria such as *Bifidobacterium*, *Lactobacillus*/*Lacticaseibacillus*, *Ruminococcus*, *Faecalibacterium*, *Roseburia*, *Anaerostipes*, and other members of the *Firmicutes* and *Actinobacteria*. This distinction is particularly important in mushroom matrices, where β-glucans, chitin, and other non-digestible polysaccharides are likely to act as the primary fermentable substrates, whereas phenolic compounds may function mainly as ecological and metabolic co-modulators. They are also linked to antioxidant and antiproliferative actions in the colon [37,38,42], and have been associated with potential benefits in metabolic syndrome, cardiovascular, and neurological conditions, often mediated through the gut-liver-brain and related axes [28]. However, the bacterial strains, genes, enzymes, and metabolite trajectories specifically involved in the biotransformation of mushroom-derived phenolics remain insufficiently mapped. Therefore, extrapolation from plant polyphenol-metabolizing consortia should be made cautiously. Future studies should combine defined mushroom phenolic fractions or phenolic-rich extracts with anaerobic fermentation models, metagenomics, metatranscriptomics, and metabolomics to identify the microbial taxa and enzymatic pathways responsible for the conversion of fungal phenolic compounds. Thus, microbial biotransformation constitutes a central mechanism linking the limited intestinal absorption of phenolic compounds to both local and systemic health effects. Although a substantial body of research exists on plant-derived phenolics, knowledge regarding the colonic biotransformation of mushroom-derived phenolic compounds remains limited. In vitro colon models applied to mushrooms such as Pleurotus ostreatus, Lentinula edodes, and Agaricus bisporus have reported the formation of metabolites including gentisic, homogentisic, and caffeic acids [43], alongside a consistent increase in *Lactobacillus*, *Bifidobacterium* and some *Ruminococcus* groups [15]. However, despite these consistent genus-level associations, the specific microbial species and enzymatic pathways responsible for the direct metabolism of mushroom-derived phenolics in the human colon remain largely unresolved. Most current studies rely on in vitro fermentation systems and taxonomic resolution at the genus level rather than precise species-specific metabolic assignments, and even reviews describing the phenolic richness of mushrooms and their antimicrobial or prebiotic potential do not yet delineate clear colonic biotransformation pathways linked to individual microbial species [44].

As described previously, most of the mechanistic evidence currently available comes from studies on plant-derived phenolic compounds, so these findings should be interpreted and extrapolated to mushroom phenolic compounds with some caution, even though similar microbial biotransformation pathways in the colon are likely to occur.

### 3.4. Mushroom-Derived Phenolic Compounds: Colonic Fate and Emerging Evidence

While microbial biotransformation of dietary phenolic compounds has been extensively characterized in plant-based matrices, evidence regarding mushroom-derived phenolic compounds remains limited. Nevertheless, the available studies suggest that mushrooms may also exert modulatory effects on the gut microbiota and undergo relevant microbial metabolism in the colon. In this context, the study performed by Andrade et al. [15] demonstrated that digested *Pleurotus djamor* powder, containing phenolic compounds such as epicatechin, gallic acid, and quercetin-3-O-glucoside, showed prebiotic-like, matrix-dependent effects in an in vitro colonic fermentation assay. Specifically, the digested mushroom increased the relative abundance of *Lactobacillus* spp./*Enterococcus* spp. (from 1.12% to 4.83%), *Bifidobacterium* spp. (from 0.59% to 1.85%), *Ruminococcus albus*/*R. flavefaciens* (from 0.37% to 1.88%), and reduced *Clostridium histolyticum* (from 2.89% to 1.22%) after 48 h of colonic fermentation. Moreover, this digested mushroom enhanced lactic acid and SCFA production and a decrease in pH. The detection of metabolites such as homovanillate and 3-hydroxyphenylacetate both before and after in vitro colonic fermentation suggests that these phenolic compounds from *Pleurotus djamor* are not completely degraded during simulated gastrointestinal digestion and may remain available in the colonic fermentation medium [15]. Complementary evidence supporting the microbial transformation of mushroom phenolic compounds has been reported in fermentation-based studies. Ayar-Sümer et al. demonstrated that lactic acid bacteria fermentation of *Lentinula edodes* and *Lactarius deliciosus* promoted the release of bound phenolics and induced substantial modifications in phenolic profiles, leading the formation of simpler phenolic metabolites and enhanced bioaccessibility. In fermented mushrooms, the phenolic compounds identified comprised several subclasses, including hydroxybenzoic acids, hydroxycinnamic acids, phenols, flavan-3-ols, flavonols, isoflavones, and stilbenes [45]. These original studies provide mushroom-specific experimental support for the gastrointestinal persistence, microbial transformation and matrix-dependent bioaccessibility of phenolic compounds. Nevertheless, they also illustrate an important limitation of the current evidence: phenolic compounds were evaluated within whole or fermented mushroom matrices rather than as purified isolated compounds. Therefore, these findings strengthen the biological plausibility of a role for mushroom-derived phenolics in microbiota-related modulation, but they do not yet establish phenolics as independent drivers of prebiotic-like effects.

## 4. Potential Prebiotic-like Contribution of Phenolic Compounds Within Mushroom Matrices

**Figure 2:**
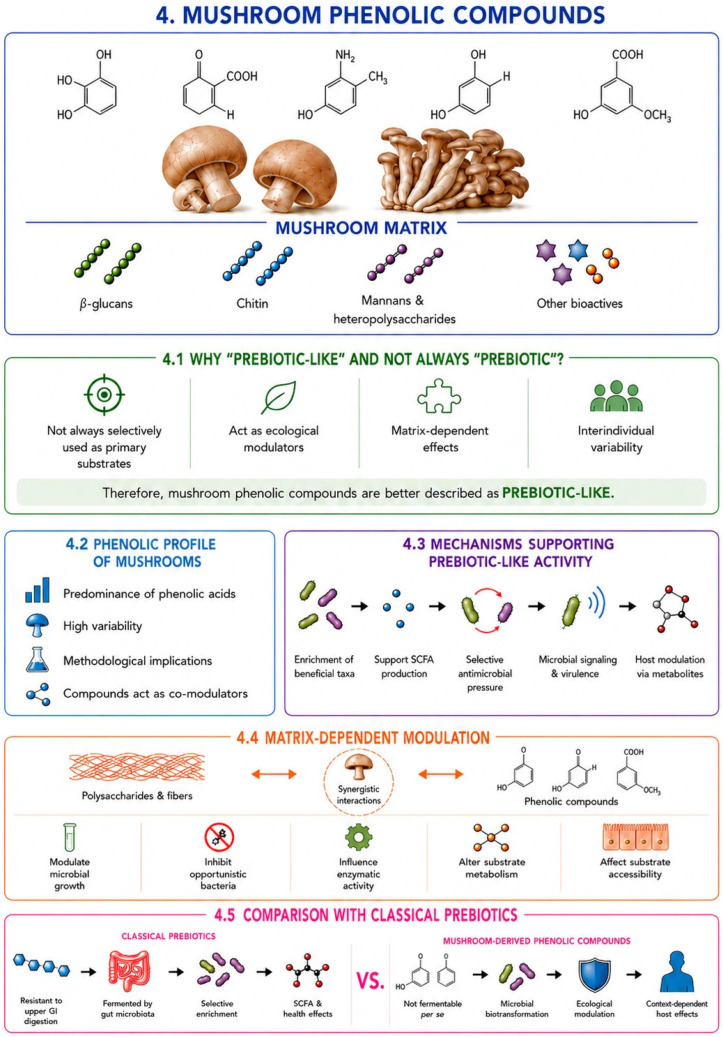
Schematic overview of the prebiotic-like effects of mushroom-derived phenolic compounds. Mushroom phenolic acids are proposed to act primarily as ecological modulators of the gut microbiota rather than as classical fermentable prebiotic substrates.

The main concepts and proposed mechanisms by which mushroom-derived phenolic compounds may contribute to prebiotic-like modulation within complex fungal matrices are summarized in Figure 2.

### 4.1. Why “Prebiotic-like” and Not Always “Prebiotic”?

The classification of polyphenols as prebiotic compounds requires caution. According to the International Scientific Association for Probiotics and Prebiotics (ISAPP), a prebiotic is defined as “a substrate that is selectively utilized by host microorganisms conferring a health benefit” [13]. This definition implies three interrelated criteria: selective microbial utilization, demonstrable use as a substrate by host-associated microorganisms, and a causal relationship between microbial modulation and measurable host benefits [46,47]. In general, phenolic compounds interact extensively with the gut microbiota, but they do not always fulfil all criteria required for classification as true prebiotics. Unlike classical prebiotics, such as inulin, fructooligosaccharides, galactooligosaccharides, resistant starch, or fungal β-glucans, mushroom-derived phenolic compounds are not consistently utilized as primary fermentable substrates or major energy sources by host microorganisms. Instead, many phenolic compounds undergo partial microbial biotransformation, generating smaller phenolic metabolites with distinct biological activities [39,40]. Therefore, mushroom phenolics should be regarded primarily as ecological modulators of the intestinal ecosystem rather than as established prebiotic substrates. Their effects may involve modulation of microbial competition, selective antimicrobial pressure, microbial enzymatic activity, redox-related interactions, host–microbe signaling, and matrix-dependent interactions with other fermentable mushroom components.

This distinction is particularly relevant for mushrooms, in which phenolic compounds coexist with β-glucans, chitin and other non-digestible fungal components that are more clearly established as fermentable substrates for gut microorganisms. Thus, any microbiota-related effect observed after mushroom intake should not be attributed to phenolic compounds alone but rather interpreted as a matrix-dependent outcome arising from the combined action of phenolic acids, fungal polysaccharides and other bioactive constituents.

Edible and medicinal mushrooms contain several phenolic constituents, predominantly low-molecular-weight phenolic acids, including gallic, protocatechuic, *p*-hydroxybenzoic, caffeic, *p*-coumaric, ferulic and vanillic acids. However, their abundance varies according to mushroom species, strain, cultivation substrate, developmental stage, environmental conditions, post-harvest processing and extraction methodology [1,48,49]. This chemical variability is relevant because different phenolic acids may differ in microbial accessibility, metabolic fate and biological activity.

However, mushrooms are also rich in non-digestible polysaccharides, β-glucans, chitin and other dietary fibers, which are more clearly established as fermentable substrates for gut microorganisms than mushroom-derived phenolic compounds themselves. Therefore, in mushroom-based interventions, microbiota-related effects should be interpreted with caution, as they may arise from the combined action of phenolic acids, β-glucans, chitin, heteropolysaccharides, terpenoids and other bioactive constituents rather than from phenolic compounds alone [50,51,52].

Evidence for selective microbial utilization of phenolic compounds remains inconsistent. Intake of phenolic-rich foods has been associated with increases in taxa such as *Bifidobacterium*, *Lactobacillus*, *Faecalibacterium* and *Akkermansia muciniphila*, but these effects vary according to compound class, dose, food matrix, intervention duration and baseline microbiota composition [27,46]. This variability is especially relevant for mushrooms, where phenolic compounds are embedded within a complex fungal matrix [30]. Interindividual variability further complicates classification. Differences in gut microbiota composition and microbial gene content influence the capacity to metabolize phenolic compounds into bioactive derivatives, giving rise to distinct microbial metabotypes. Consequently, two individuals consuming the same phenolic-rich mushroom product may generate different microbial metabolites and physiological responses [29]. In this context, microbial-derived phenolic metabolites may represent useful biomarkers of response in future stratified clinical trials. Urolithins provide a well-established example of microbiota-dependent polyphenol metabotypes, although they are mainly associated with ellagitannin- and ellagic acid-rich foods and should not be considered mushroom-specific metabolites. For mushroom-derived phenolics, more relevant candidate biomarkers may include hydroxybenzoic acids, hydroxyphenylacetic acids, hydroxyphenylpropionic acids, benzoic acid derivatives, and hippuric acid/hippurate-related metabolites derived from the microbial transformation of low-molecular-weight phenolic acids. Future studies should therefore combine microbiome sequencing with targeted and untargeted metabolomics in feces, plasma and urine to identify responder/non-responder phenotypes, determine whether baseline microbiota predicts phenolic biotransformation, and clarify links between microbial metabolites and host outcomes [30,53,54].

Therefore, the term “prebiotic-like” is more appropriate than “prebiotic” for mushroom-derived phenolic compounds. It recognizes their potential to modulate gut microbiota composition and metabolic activity, while avoiding the assumption that they consistently fulfil the strict criteria of selective substrate utilization and causal health benefit established for classical prebiotics [30,46].

### 4.2. Phenolic Profile of Mushrooms: Predominance of Phenolic Acids

In plant-derived foods, the term polyphenols commonly includes flavonoids, stilbenes, lignans, tannins and phenolic acids. In mushrooms, however, the phenolic fraction reported in the literature is generally dominated by low-molecular-weight phenolic acids rather than by complex flavonoid-type polyphenols. Original chromatographic studies using HPLC-DAD, HPLC-DAD-ESI/MS or LC-MS approaches have identified phenolic acids as recurrent constituents of edible and medicinal mushrooms, including hydroxybenzoic acid derivatives such as gallic, protocatechuic, *p*-hydroxybenzoic, gentisic, vanillic and syringic acids, and hydroxycinnamic acid derivatives such as caffeic, *p*-coumaric, ferulic, sinapic and cinnamic acids [46,55,56,57].

This predominance of phenolic acids has been reported across different mushroom species, including wild and cultivated taxa. For example, Barros et al. analysed sixteen Portuguese wild mushroom species by HPLC-DAD-ESI/MS and reported several phenolic acids, including protocatechuic, *p*-hydroxybenzoic and *p*-coumaric acids, whereas flavonoids were not detected in the analysed samples [58]. Palacios et al. also investigated phenolic compounds in cultivated and wild edible mushrooms and identified individual phenolic compounds contributing to antioxidant activity [56]. Similarly, studies on wild edible mushrooms from different geographical origins have reported species-dependent profiles of phenolic acids, reinforcing the relevance of mushroom species, origin and analytical methodology in determining the phenolic composition [55,57,59].

The abundance and profile of these compounds vary markedly according to mushroom species, strain, cultivation substrate, developmental stage, environmental conditions, post-harvest processing and extraction methodology [57,59,60]. This variability is important because phenolic acids differ in microbial accessibility, antimicrobial potency, chemical stability and capacity for microbial transformation. Consequently, mushroom-derived phenolic compounds should not be treated as a homogeneous class.

This distinction also has methodological implications. In many mushroom studies, microbiota-related effects are evaluated using whole mushroom powders, crude extracts or polysaccharide-rich fractions. Under these conditions, it is difficult to attribute effects specifically to phenolic compounds, because mushrooms also contain β-glucans, chitin, mannans, heteropolysaccharides, proteins, terpenoids, ergosterol derivatives and other bioactive constituents [1,51,52]. For this reason, mushroom phenolic compounds should be interpreted as co-modulators within a broader fungal matrix rather than as isolated prebiotic substrates.

### 4.3. Potential Mechanisms Supporting Prebiotic-like Modulation

Several mechanisms may explain how phenolic compounds contribute to the prebiotic-like modulation observed in phenolic-containing foods and mushroom matrices. These mechanisms are not mutually exclusive and may operate simultaneously within the intestinal ecosystem.

However, in the specific case of mushrooms, most mechanistic evidence remains indirect, as many studies use whole mushroom powders, crude extracts or polysaccharide-rich fractions rather than purified mushroom-derived phenolic acids. Therefore, microbiota-related outcomes should be interpreted as matrix-associated effects in which phenolics may act as co-modulators alongside β-glucans, chitin, heteropolysaccharides and other fungal constituents.

Therefore, throughout this section, the term “mushroom-derived phenolic compounds” should be interpreted with caution. Unless studies used purified phenolic acids or standardized phenolic-enriched fractions, microbiota-related outcomes are best understood as matrix-associated effects in which phenolics may act as co-modulators alongside β-glucans, chitin, heteropolysaccharides and other fungal constituents, rather than as isolated drivers of prebiotic-like activity.

First, phenolic compounds may contribute to the enrichment of bacterial taxa associated with intestinal and metabolic homeostasis. Dietary phenolic-rich interventions have been associated with increases in *Bifidobacterium*, *Lactobacillus*, *Faecalibacterium* and *Akkermansia muciniphila*, although responses vary according to compound class, dose, food matrix, duration of intervention and baseline microbiota composition [61,62]. In an original experimental study, Anhê et al. showed that a polyphenol-rich cranberry extract increased *Akkermansia* abundance and improved metabolic parameters in high-fat diet-fed mice [63]. Although this study was not performed with mushroom-derived phenolic compounds, it provides mechanistic support for the concept that poorly absorbed phenolic compounds can reshape gut microbial communities and influence host metabolism.

Second, phenolic compounds may indirectly support SCFA production through microbial cross-feeding. Phenolic acids are not major fermentable substrates in the same way as dietary fibers, but their microbial transformation can generate smaller metabolites that influence microbial metabolic networks [47]. Original in vitro fermentation studies have shown that dietary fibers can modify the microbial catabolism of flavonoids into phenolic acids, indicating that the interaction between phenolic compounds and fermentable substrates can shape microbial metabolism [64]. In mushrooms, this mechanism is particularly relevant because phenolic acids coexist with β-glucans, chitin and other fungal polysaccharides, which provide more direct fermentable substrates for SCFA production [50,51,52].

Third, mushroom-derived phenolic acids may contribute to selective antimicrobial pressure. This is one of the best-supported mechanisms using mushroom-specific original evidence. Alves et al. evaluated phenolic compounds identified in wild mushrooms and showed antimicrobial activity against Gram-positive and Gram-negative bacteria, including resistant strains. In that study, protocatechuic acid and 2,4-dihydroxybenzoic acid showed particularly relevant antimicrobial activity [60,65]. Such selective antimicrobial effects may indirectly favour beneficial microbial groups by reducing ecological competition from opportunistic taxa, although this mechanism still requires confirmation in gut microbiota models.

Fourth, phenolic compounds may modulate microbial signalling and virulence-related behaviours. Original studies using bacterial quorum-sensing biosensors have shown that several phenolic compounds can interfere with quorum sensing, biofilm formation and virulence-related pathways [61]. This mechanism is relevant because it suggests that phenolic compounds may modulate microbiota function without necessarily acting as classical fermentable substrates. In mushrooms, this remains underexplored, but it is biologically plausible given the presence of phenolic acids with antimicrobial and enzyme-modulating activity.

Finally, phenolic compounds may influence host intestinal physiology through microbial-derived metabolites. Gut microbiota can transform phenolic compounds into smaller phenolic acids and related metabolites, which may exert anti-inflammatory, antioxidant and metabolic effects [47]. However, the causal chain linking mushroom-derived phenolic acids, microbial biotransformation, microbiota modulation and host outcomes remains insufficiently demonstrated, particularly in human studies. Therefore, mushroom-derived phenolic compounds should currently be considered prebiotic-like ecological modulators rather than established prebiotic substrates. To avoid overinterpretation, mechanistic claims in this section should be viewed according to the level of evidence available. Mechanisms such as microbial biotransformation, selective antimicrobial pressure and changes in phenolic bioaccessibility are supported by original mushroom-related experimental studies, although mostly using whole mushrooms, fermented mushrooms or phenolic-containing fungal matrices. In contrast, broader mechanisms involving host metabolic regulation, SCFA-related responses and intestinal barrier effects are partly extrapolated from the wider dietary polyphenol literature and should therefore be considered biologically plausible but not yet fully demonstrated for isolated mushroom-derived phenolic compounds.

### 4.4. Matrix-Dependent Modulation: Synergy with β-Glucans, Chitin and Fungal Polysaccharides

The prebiotic-like relevance of mushroom-derived phenolic compounds is best understood within the mushroom food matrix. Mushrooms are not merely sources of isolated phenolic acids; they are complex fungal matrices containing β-glucans, chitin, mannans, heteropolysaccharides, proteins, minerals, sterols and other bioactive constituents. This matrix-dependent interpretation is essential because the most direct evidence for prebiotic activity in mushrooms relates to non-digestible polysaccharides and dietary fiber rather than to phenolic compounds alone [1,51,52].

Original studies support this view. In a randomized crossover study in healthy adults, Hess et al. evaluated the effects of *Agaricus bisporus* mushroom consumption on gut health markers and reported effects on gastrointestinal tolerance, fecal microbiota and SCFA production, suggesting that whole mushroom intake can influence gut microbial metabolism [66]. Similarly, Solano-Aguilar et al. showed in pigs that dietary Agaricus bisporus modified intestinal microbiota composition and host immune responses, supporting the potential prebiotic activity of whole mushrooms as a complex food matrix rather than a single-compound intervention [67].

Evidence from in vitro fermentation studies further reinforces the fermentability of mushroom-derived polysaccharides. Duan et al. evaluated *Agaricus bisporus* polysaccharides using human fecal microbiota fermentation and showed that these polysaccharides modified microbial composition and metabolite production, including SCFAs [68]. Other in vitro studies using mushroom-derived polysaccharides or fungal polysaccharide fractions have similarly shown decreases in pH, increased SCFA generation and shifts in bacterial communities, indicating that fungal polysaccharides can serve as substrates for gut microbial fermentation [69,70].

Within this framework, mushroom-derived phenolic compounds should be interpreted as co-modulators rather than dominant fermentable substrates. Phenolic acids may influence microbial growth, inhibit opportunistic bacteria, modulate microbial enzymatic activity and alter the metabolic fate of other mushroom-derived substrates. Conversely, β-glucans, chitin and heteropolysaccharides may influence the colonic delivery, retention time and microbial accessibility of phenolic acids through physicochemical interactions within the fungal matrix. Evidence from dietary fiber–polyphenol research indicates that phenolic compounds may associate with polysaccharide-rich matrices through non-covalent interactions, including hydrogen bonding, hydrophobic interactions, van der Waals forces and, depending on the chemical environment, electrostatic interactions [71,72]. These interactions may reduce phenolic release in the upper gastrointestinal tract, increase retention within the indigestible matrix, and promote a more gradual release during colonic fermentation, where microbial enzymes progressively degrade the fiber network and liberate bound phenolics. In mushrooms, similar matrix-dependent interactions may occur between phenolic acids and fungal polysaccharides such as β-glucans, chitin and heteropolysaccharides, potentially shaping colonic release kinetics, microbial accessibility and downstream biotransformation. Thus, the prebiotic-like activity of mushrooms may emerge from coordinated interactions between fermentable fungal polysaccharides and phenolic compounds embedded within the same matrix.

For clarity, the mechanistic contribution of each mushroom component should be interpreted separately. β-glucans, chitin and other fungal polysaccharides are the most plausible drivers of direct fermentative effects, including pH reduction, SCFA production and enrichment of saccharolytic bacterial groups. In contrast, mushroom-derived phenolic compounds are more appropriately considered ecological and metabolite-mediated co-modulators, with potential roles in microbial biotransformation, selective antimicrobial pressure, modulation of microbial enzymatic activity, redox-related effects and downstream effects on epithelial barrier integrity and inflammatory signaling. Therefore, microbiota-related effects observed after whole mushroom intake or crude mushroom extracts should not be attributed exclusively to phenolic compounds, but rather interpreted as the result of overlapping contributions from fermentable fungal polysaccharides, phenolic compounds and the broader mushroom matrix.

This interpretation is important for avoiding overstatement. When whole mushrooms, mushroom powders or crude extracts alter gut microbiota composition, these effects should not automatically be attributed to phenolic compounds. Instead, they should be described as matrix-dependent outcomes arising from the combined action of phenolic acids, β-glucans, chitin, heteropolysaccharides and other fungal bioactives. Future studies comparing whole mushroom preparations, polysaccharide-rich fractions and phenolic-enriched fractions are needed to determine the specific contribution of mushroom-derived phenolic compounds to gut microbiota modulation.

### 4.5. Comparison with Classical Prebiotics

Classical prebiotics, including inulin, fructooligosaccharides (FOSs), galactooligosaccharides (GOSs) and resistant starch, are defined by their resistance to digestion in the upper gastrointestinal tract and selective utilization by gut microorganisms, resulting in measurable health benefits [14]. Their mechanisms are primarily linked to direct saccharolytic fermentation, enrichment of beneficial microbial taxa and production of SCFAs.

This mode of action has been supported by human intervention studies. For example, FOS supplementation increased the relative abundance of *Bifidobacterium* and *Lactobacillus* in a prospective randomized, double-blind, placebo-controlled trial [73]. Similarly, resistant starch supplementation has been shown to alter gut microbiome composition and SCFA-related outcomes in adults, supporting its classification as a fermentable prebiotic substrate [74]. These examples illustrate the more direct and substrate-driven mechanism of classical prebiotics.

Mushroom-derived phenolic compounds differ substantially from these substrates. They are not fermentable carbohydrates and should not be presented as equivalent to inulin, FOS, GOS or resistant starch. Rather, their activity is more consistent with ecological modulation: they may be transformed by gut microbiota, exert selective antimicrobial pressure, influence microbial enzymatic activity and contribute to host–microbe signalling. Therefore, their effects are less predictable and more dependent on chemical structure, dose, food matrix and baseline microbiota composition.

In mushrooms, this distinction is especially important because the strongest prebiotic candidates are fungal polysaccharides and dietary fibers, rather than phenolic acids. Original studies with *Agaricus bisporus* support this distinction. Hess et al. reported that whole mushroom consumption in healthy adults influenced fecal microbiota composition and gut health markers, but this effect was observed with the whole mushroom matrix rather than isolated phenolic compounds. In vitro fermentation studies also show that Agaricus bisporus polysaccharides can be metabolized by human gut microbiota, increasing SCFA production and modifying microbial composition [66].

Accordingly, mushroom-derived phenolic compounds should be regarded as prebiotic-like modulators acting within a fungal prebiotic matrix, rather than as classical prebiotics. Their contribution is likely complementary: they may shape microbial ecology, modulate antimicrobial pressure and influence the metabolic fate of fungal polysaccharides, while β-glucans, chitin and other non-digestible carbohydrates provide the principal fermentable substrates.

## 5. Impact on Gut Health and Host Metabolism

### 5.1. SCFA Production and Microbial Metabolites

Mushroom-derived polyphenols may influence intestinal health and host metabolism through microbiota-dependent mechanisms. In the intestinal context, these compounds should not be understood solely as direct antioxidant or anti-inflammatory agents, but rather as molecules capable of interacting with the microbial ecosystem, while being simultaneously modulated and transformed by it. This bidirectional interaction enables the formation of lower-molecular-weight phenolic metabolites, which may be more bioavailable and biologically active, exert local effects on the intestinal mucosa and, after absorption, contribute to systemic responses. Thus, fungal polyphenols may act as prebiotic-like modulators, promoting a more balanced intestinal environment and contributing to mucosal homeostasis [75].

**Figure 3:**
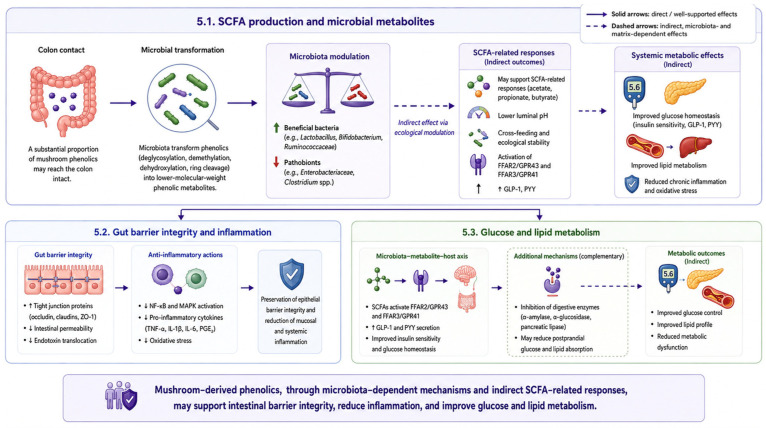
Proposed microbiota-dependent mechanisms linking mushroom-derived phenolic compounds to intestinal and metabolic outcomes. After reaching the colon, mushroom phenolics may undergo microbial biotransformation into lower-molecular-weight metabolites that modulate microbial ecology, redox balance and host–microbe signalling. Their effects on short-chain fatty acid (SCFA)-related responses are mainly indirect, occurring through ecological modulation of fermentative and SCFA-producing bacteria rather than direct SCFA generation from phenolics. These interactions may contribute to gut barrier integrity, inflammatory regulation, glucose homeostasis and lipid metabolism.

The importance of this interaction becomes evident when considering the low availability of polyphenols. Only a fraction is absorbed in the upper gastrointestinal tract, whereas a substantial proportion may reach the colon almost unchanged, becoming available for microbial metabolism. In this compartment, the gut microbiota can transform polyphenols through reactions such as deglycosylation, demethylation, dehydroxylation and aromatic ring cleavage, generating lower-molecular-weight phenolic compounds that may be more absorbable and biologically active. Therefore, the bioactivity of mushroom-derived polyphenols depends not only on their initial concentration in the extract, but also on their digestive stability, their release from the fungal matrix, their colonic biotransformation and the metabolic capacity of the resident microbiota [12]. These interconnected mechanisms are summarized in Figure 3, which provides a conceptual overview of how mushroom-derived phenolic compounds may link microbial biotransformation, microbiota modulation, microbial metabolite production, intestinal barrier regulation, inflammatory responses, and host metabolic effects.

This dynamic has been observed in in vitro studies with fungal matrices subjected to simulated gastrointestinal and colonic conditions. Vamanu et al. evaluated mycelia of Pleurotus ostreatus and Lentinula edodes, as well as dried basidiomes of *Agaricus brunnescens*, using a system simulating the different regions of the human colon. The authors found that, during colonic fermentation, differential release and availability of phenolic compounds occurred, with phenolic acids such as gentisic acid, homogentisic acid and small amounts of caffeic acid being identified. Moreover, polyphenol content was shown to correlate with antioxidant status and microbial composition, particularly with the presence of *Lactobacillus* and *Bifidobacterium*, suggesting a relationship between the availability of fungal phenolic compounds, local antioxidant activity and the modulation of beneficial bacteria [43]. Further evidence was provided by Vamanu and Pelinescu (2017) [76], who analyzed the effect of different edible mushroom species, including *Boletus edulis*, *Boletus pinophilus*, *Boletus aureus*, *Armillaria mellea*, *Lactarius piperatus* and *Pleurotus eryngii*, on microbiotas from clinically healthy individuals, individuals with nutritional disorders and individuals with cardiovascular disease. The study showed that simulated mushroom consumption altered the microbial profile in a manner dependent on the fungal species and on the physiometabolic status of the donor microbiota, affecting *Lactobacillus*, *Bifidobacterium* and *Enterobacteriaceae*. Furthermore, the microbiota composition of the individuals contributed to changes in the phenolic acid profile through fermentative activity, with gallic acid emerging as the most stable phenolic compound among the tested species, particularly after exposure to *Boletus* species and, to a lesser extent, to *Pleurotus eryngii*. In the group with nutritional disorders, the lower availability of gallic acid was associated with a higher presence of coliforms, suggesting that the stability and availability of phenolic compounds in the colon may be associated with a less favorable microbial profile, compatible with dysbiotic states [76].

Modulation of the microbiota by mushroom-derived phenolic compounds may also influence the profile of microbial metabolites. Among these, SCFAs, including acetate, propionate and butyrate, are particularly relevant for intestinal and metabolic health. These metabolites are mainly produced through the microbial fermentation of non-digestible substrates present in the fungal matrix, particularly β-glucans, chitin, and other polysaccharides, and should therefore not be presented as direct products of polyphenol metabolism. Instead, mushroom-derived polyphenols may contribute indirectly to SCFA regulation by reshaping the colonic ecological environment. After reaching the colon, these compounds can undergo microbial biotransformation into smaller phenolic metabolites, which may exert selective antimicrobial or growth-modulating effects, contribute to changes in redox conditions and, indirectly, in luminal pH, and affect microbial competition and cross-feeding interactions. Through these mechanisms, phenolic compounds may favor the persistence or metabolic activity of fermentative and SCFA-producing bacteria, including butyrate-producing taxa, while limiting the expansion of opportunistic or proteolytic bacteria [12].

A more recent example is provided by Andrade et al. (2024) [15], whose study illustrates the functional relevance of the interaction between *Pleurotus djamor*, the microbiota, and colonic metabolites. After simulated gastrointestinal digestion and in vitro colonic fermentation, digested *P. djamor* powder, containing phenolic compounds such as epicatechin, gallic acid and quercetin-3-O-glucoside, promoted a favorable fermentative profile, with increases in *Lactobacillus* spp./*Enterococcus* spp., *Bifidobacterium* spp. and *Ruminococcus albus*/*R. flavefaciens*, accompanied by a reduction in *Clostridium histolyticum*. In addition, in vitro colonic fermentation increased the production of lactic acid and SCFAs, particularly propionate and butyrate, whose levels increased approximately 6.5- and 3.7-fold, respectively, after 48 h of fermentation, in parallel with a significant reduction in pH. Although these effects cannot be attributed exclusively to phenolic compounds, this study supports the concept that phenolic-containing mushroom matrices may create a colonic environment favorable to saccharolytic fermentation and SCFA production [15].

Thus, the available evidence suggests that mushroom-derived polyphenols, although not the main fermentable substrates responsible for SCFA production, may participate in the regulation of the colonic environment through their microbial biotransformation, the formation of bioactive phenolic metabolites and the indirect modulation of bacteria involved in the production of metabolites relevant to intestinal and metabolic homeostasis.

### 5.2. Gut Barrier Integrity and Inflammation

The preservation of the intestinal barrier represents one of the main mechanisms through which mushroom-derived polyphenols may contribute to host health. Under dysbiotic conditions, a reduction in the abundance of beneficial bacteria and protective metabolites, increased epithelial permeability and the translocation of pro-inflammatory bacterial components, such as lipopolysaccharides, may trigger persistent immune activation. This scenario promotes chronic low-grade inflammation, a phenomenon associated not only with intestinal disorders but also with systemic metabolic disturbances.

From a functional perspective, SCFAs constitute one of the main links between the microbiota and the intestinal barrier. Butyrate is particularly important because it represents an essential energy source for colonocytes and contributes to the maintenance of epithelial barrier integrity by promoting the expression and organization of junctional proteins, such as occludin, claudins and ZO-1. Acetate and propionate, in turn, participate in cross-feeding interactions and in the regulation of the luminal environment, contributing to pH reduction and to the ecological stability of the colon [77,78]. In this context, phenolic metabolites formed in the colon may exert local antioxidant activity, reduce the accumulation of reactive oxygen species in the intestinal mucosa and contribute to the preservation of epithelial integrity. Beyond barrier protection, fungal polyphenols and their microbial metabolites may interfere with inflammatory pathways relevant to intestinal homeostasis. The modulation of pathways such as NF-κB and MAPK may contribute to the downregulation of pro-inflammatory cytokines and other mediators associated with mucosal inflammation. This action is particularly relevant in situations in which oxidative stress, dysbiosis and epithelial dysfunction reinforce one another, as occurs in intestinal inflammatory processes and in metabolic states characterized by systemic low-grade inflammation [12]. Experimental support for this hypothesis comes from studies using phenolic compounds isolated from mushrooms and phenolic extracts from edible species. Liu et al. (2018) [79], for example, identified farnesyl phenolic compounds from *Ganoderma duripora* with anti-inflammatory activity in LPS-stimulated RAW 264.7 macrophages. These compounds reduced pro-inflammatory mediators, including TNF-α, IL-1β, IL-6 and PGE_2_, and ganoduriporol A was shown to inhibit COX-2 expression through modulation of the MAPK and NF-κB pathways, providing mechanistic support for the hypothesis that mushroom-derived phenolics may attenuate inflammatory responses induced by bacterial stimuli [79].

In line with this evidence, Taofiq et al. (2015) [80] demonstrated that ethanolic extracts of edible mushrooms, including *Pleurotus ostreatus*, *Macrolepiota procera*, *Boletus impolitus* and, exhibited anti-inflammatory activity in LPS-stimulated RAW 264.7 macrophages, with inhibition of nitric oxide production. This activity was associated with the presence of phenolic acids, particularly cinnamic acid, *p*-coumaric acid and *p*-hydroxybenzoic acid, as well as their methylated and glucuronidated derivatives, suggesting that phenolics present in mushrooms and their metabolites may modulate inflammatory responses [80].

Therefore, mushroom-derived polyphenols may contribute to intestinal health through a combination of antioxidant, anti-inflammatory and microbiota-dependent effects. These mechanisms may favor epithelial barrier integrity, limit endotoxin translocation and reduce local inflammatory stimuli that, when persistent, may affect systemic metabolism.

### 5.3. Glucose and Lipid Metabolism

The effects of mushroom-derived polyphenols may also extend beyond the intestine, influencing host metabolism through the microbiota–metabolite–host axis. After colonic biotransformation, lower-molecular-weight phenolic metabolites may be absorbed and exert systemic effects, particularly by modulating oxidative stress, metabolic inflammation and pathways associated with glucose and lipid homeostasis. These mechanisms are particularly relevant in the context of obesity, type 2 diabetes, metabolic syndrome and cardiovascular disease, in which gut dysbiosis, metabolic endotoxemia, chronic low-grade inflammation and alterations in glycolipid metabolism are frequently interconnected [12,78]. Within this axis, SCFAs produced during colonic fermentation can act as signaling molecules through the receptors FFAR2/GPR43 and FFAR3/GPR41, which are involved in communication between the microbiota, enteroendocrine cells, adipose tissue, the liver and the immune system. Activation of these receptors has been associated with the secretion of gut hormones, such as GLP-1 and PYY, which participate in the regulation of satiety, intestinal motility, glucose homeostasis and insulin sensitivity. Tolhurst et al. (2012) [81] demonstrated that SCFAs stimulate GLP-1 secretion through mechanisms mainly dependent on FFAR2, reinforcing the relevance of these metabolites in glycemic regulation. Complementarily, Lee et al. (2024) [82] highlighted that GPR41 and GPR43 act as SCFA sensors and participate in processes associated with obesity, diabetes, inflammation and cardiovascular disease [81,82].

The relationship between microbial metabolites and glucose metabolism is also supported by clinical and preclinical evidence on SCFAs. A systematic review and meta-analysis on SCFA and insulin sensitivity showed that post-intervention increases in SCFA levels are associated with lower fasting insulin concentrations, supporting the link between colonic fermentation, microbial metabolites and improved insulin sensitivity [83]. In the context of mushrooms, these mechanisms are relevant because fungal polyphenols may indirectly modulate the fermentative profile of the microbiota, while their phenolic metabolites may contribute to reducing oxidative stress and low-grade inflammation, two central processes in insulin resistance and metabolic dysfunction.

Beyond SCFA-mediated signaling, mushroom extracts rich in phenolic compounds have been associated with the modulation of digestive enzymes involved in glucose and lipid metabolism. For example, in vitro studies with fruiting bodies of Agaricus bisporus showed inhibitory activity against α-amylase, α-glucosidase and pancreatic lipase, enzymes relevant to the digestion of carbohydrates and lipids [84]. Although these effects cannot be attributed exclusively to polyphenols, this type of evidence supports the hypothesis that mushroom bioactive compounds, including phenolics, may contribute to the postprandial modulation of glucose and lipid absorption.

Thus, the impact of mushroom-derived polyphenols on glucose and lipid metabolism should be understood as the result of complementary mechanisms. Phenolic metabolites derived from colonic biotransformation may contribute to the reduction in oxidative stress and metabolic inflammation, while the indirect modulation of microbial metabolites, such as SCFAs, may influence metabolic receptors, gut hormones and insulin sensitivity. Additionally, the potential interference of fungal extracts rich in phenolic compounds with digestive enzymes suggests a possible contribution to the postprandial regulation of glucose and lipid absorption. Taken together, although specific evidence using purified mushroom-derived polyphenols remains limited, these compounds emerge as functional modulators of the microbiota–metabolite–metabolism axis.

## 6. Therapeutic Relevance in Chronic Disorders

**Figure 4:**
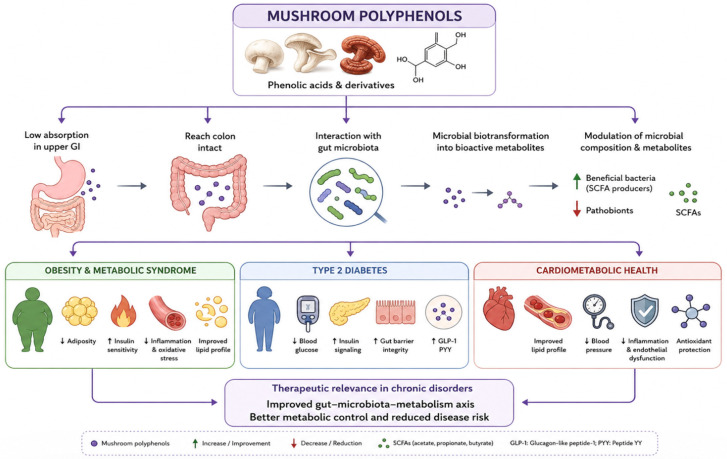
Proposed role of mushroom-derived phenolic compounds in chronic metabolic disorders. Mushroom phenolics may undergo microbial biotransformation and modulate gut microbial ecology, intestinal barrier integrity, oxidative stress, inflammation, SCFA-related signaling, glucose homeostasis and lipid metabolism. These mechanisms remain mainly mechanistic and require validation in well-designed human studies.

The interaction between mushroom-derived phenolic compounds, gut microbiota, and host metabolism has generated increasing interest regarding their potential therapeutic relevance in chronic diseases. Disorders such as obesity, metabolic syndrome, type 2 diabetes, and cardiometabolic diseases are now recognized as multifactorial conditions strongly associated with chronic low-grade inflammation, oxidative stress, intestinal barrier dysfunction, microbial dysbiosis, and altered microbial metabolite production. Within this framework, dietary compounds capable of modulating the microbiota–metabolite–host axis have emerged as promising targets for nutritional and preventive strategies. These compounds may influence metabolic pathways associated with insulin sensitivity, lipid metabolism, adipose tissue dysfunction, endothelial integrity, and immune regulation. The potential therapeutic relevance of mushroom-derived phenolic compounds in chronic disorders is therefore best understood as an integrated, multi-level process involving microbial biotransformation, modulation of gut microbial ecology, intestinal barrier regulation, inflammatory control, and systemic metabolic signaling. These interconnected mechanisms are summarized in Figure 4, which provides a conceptual overview of how mushroom phenolics may contribute to obesity, metabolic syndrome, type 2 diabetes, and cardiometabolic health through both microbiota-dependent and host-mediated pathways. 

### 6.1. Obesity and Metabolic Syndrome 

Obesity and metabolic syndrome are chronic multifactorial conditions characterized by excessive adiposity, insulin resistance, dyslipidemia, hypertension, low-grade inflammation, oxidative stress, and gut microbiota dysbiosis. These alterations are closely interconnected: adipose tissue expansion promotes macrophage infiltration and inflammatory cytokine release, while impaired intestinal barrier function and metabolic endotoxemia further aggravate systemic inflammation and insulin resistance [85,86]. Within this pathophysiological framework, mushroom-derived phenolic compounds should not be considered only as antioxidant molecules, but rather as bioactive metabolites capable of modulating inflammatory, redox, microbial, and metabolic pathways involved in adipose tissue dysfunction and cardiometabolic impairment. As illustrated in Figure 4, these effects may involve the combined modulation of gut microbial composition, microbial metabolite production, intestinal barrier function, metabolic endotoxemia, adipose tissue inflammation, and host energy-regulating pathways.

Edible and medicinal mushrooms contain several phenolic acids with potential relevance for obesity-related metabolic disturbances, including protocatechuic acid, gallic acid, caffeic acid, ferulic acid, *p*-coumaric acid, *p*-hydroxybenzoic acid, syringic acid, chlorogenic acid, and cinnamic acid derivatives [1,2,19]. These compounds have been reported in species such as *Agaricus bisporus*, *Pleurotus ostreatus*, *Lentinula edodes*, *Ganoderma lucidum*, *Hericium erinaceus*, *Morchella esculenta*, *Boletus edulis*, and other wild edible mushrooms [3,4,5]. Among these phenolics, protocatechuic acid, caffeic acid, ferulic acid, and chlorogenic acid are especially relevant because they have been associated with modulation of adipogenesis, lipid metabolism, mitochondrial function, oxidative stress, insulin sensitivity, and inflammatory signaling [87,88,89].

Protocatechuic acid is one of the most biologically relevant phenolic metabolites in this context. Experimental evidence indicates that it can attenuate adipocyte inflammation, improve mitochondrial dysfunction, and regulate AMPK/Akt-dependent signaling pathways [87,88,89]. These effects are directly relevant to obesity and metabolic syndrome, since hypertrophic adipose tissue is characterized by mitochondrial stress, increased production of TNF-α, IL-6, and other pro-inflammatory mediators, reduced adiponectin secretion, and impaired insulin signaling [85]. In inflamed adipocyte models, protocatechuic acid has been shown to reduce inflammatory responses and improve mitochondrial respiration, suggesting a role in limiting obesity-associated adipose tissue dysfunction. 

Caffeic acid is another mushroom-associated phenolic acid with relevance for metabolic syndrome. It has been associated with anti-obesity, anti-inflammatory, antioxidant, antihyperglycemic, antihypertensive, and lipid-modulating effects [88,90]. In diet-induced obesity models, caffeic acid reduced body weight gain and fat accumulation, improved lipid profiles, increased energy expenditure, and partially restored gut microbiota imbalance. Importantly, these effects were accompanied by enrichment of metabolically beneficial and butyrate-producing bacteria, suggesting that caffeic acid may act through both direct host metabolic pathways and microbiota-dependent mechanisms [88].

Ferulic acid may also contribute to the metabolic effects attributed to mushroom phenolics. As a hydroxycinnamic acid, it has been associated with antioxidant and anti-inflammatory activity, improvement of lipid metabolism, and modulation of insulin sensitivity [91]. In obesity and metabolic syndrome, ferulic acid is particularly relevant because oxidative stress and chronic inflammatory signaling impair insulin receptor activity, mitochondrial function, and lipid handling in adipose tissue and liver [85,89]. Together with protocatechuic acid, ferulic acid may therefore contribute to attenuation of adipogenesis-associated inflammation and metabolic stress [89].

Chlorogenic acid, although more extensively studied in coffee and plant-derived foods than in mushrooms, has also been detected in some mushroom species and has strong mechanistic relevance for metabolic syndrome [2,92]. It has been associated with reduced body weight gain, improved glucose tolerance, enhanced insulin sensitivity, modulation of gut microbiota composition, reinforcement of intestinal barrier function, and attenuation of metabolic endotoxemia in experimental models [92]. However, when discussing chlorogenic acid in the context of mushrooms, it is important to state that most clinical and mechanistic evidence comes from non-mushroom dietary sources.

The gut microbiota provides a key mechanistic bridge between mushroom phenolics and obesity-related metabolic outcomes. Many phenolic compounds are poorly absorbed in the upper gastrointestinal tract and may reach the colon, where microbial enzymes transform them into smaller phenolic metabolites with greater bioavailability and biological activity [9]. These metabolites can influence gut microbial ecology, favoring bacteria involved in SCFA production while reducing taxa associated with endotoxemia and inflammatory tone [9,93]. This is particularly relevant in obesity, where reduced microbial diversity, impaired gut barrier function, and increased circulating lipopolysaccharide contribute to chronic inflammation and insulin resistance [86,93].

At the host level, mushroom-associated phenolic compounds may modulate several pathways central to metabolic syndrome. AMPK activation promotes fatty acid oxidation, enhances insulin sensitivity, and inhibits lipogenesis [87,94]. Suppression of NF-κB signaling reduces the expression of pro-inflammatory mediators such as TNF-α, IL-6, and IL-1β [89]. Activation of Nrf2-dependent antioxidant responses may protect against obesity-associated oxidative damage, while regulation of PPAR-related pathways may influence adipocyte differentiation, lipid storage, and systemic lipid homeostasis [87,89,94]. Thus, the therapeutic relevance of mushroom phenolics lies in their capacity to target multiple mechanisms simultaneously, including oxidative stress, inflammation, adipose tissue dysfunction, gut barrier impairment, microbial metabolism, and host energy regulation.

Nevertheless, clinical evidence specifically attributing anti-obesity or anti-metabolic syndrome effects to isolated mushroom-derived phenolic compounds remains limited. Most human studies evaluate whole mushrooms rather than purified phenolic fractions. This is a major limitation because mushrooms also contain β-glucans, chitin, ergothioneine, sterols, vitamins, minerals, and other bioactive compounds that may independently or synergistically influence metabolic outcomes. A systematic review of human studies reported that mushroom consumption may reduce triglycerides and high-sensitivity C-reactive protein, but evidence for other cardiometabolic endpoints remains inconsistent and often limited by study quality [16]. Therefore, mushroom phenolics should currently be regarded as promising mechanistic contributors rather than established therapeutic agents for obesity and metabolic syndrome.

### 6.2. Type 2 Diabetes

Type 2 diabetes mellitus is a complex metabolic disorder characterized by insulin resistance, impaired pancreatic β-cell function, chronic low-grade inflammation, oxidative stress, dysregulated lipid metabolism, altered incretin secretion, and gut microbiota dysbiosis [95,96]. Increasing evidence indicates that the pathogenesis of type 2 diabetes is influenced by bidirectional interactions between diet, intestinal microbiota, microbial metabolites, intestinal barrier function, and host metabolic signaling [9,93,96]. Within this framework, mushroom-derived phenolic compounds are biologically plausible candidates for modulating glucose homeostasis, although direct clinical validation remains limited. In this context, Figure 4 highlights the potential contribution of mushroom-derived phenolics to glucose regulation through microbial biotransformation, SCFA-related signaling, attenuation of oxidative stress, inflammatory control, and modulation of insulin-related pathways.

Phenolic acids commonly detected in edible and medicinal mushrooms include protocatechuic acid, gallic acid, caffeic acid, ferulic acid, p-coumaric acid, syringic acid, chlorogenic acid, and p-hydroxybenzoic acid. These compounds have been identified in *Agaricus bisporus*, *Pleurotus ostreatus*, *Lentinula edodes*, *Ganoderma lucidum*, *Hericium erinaceus*, and several wild mushrooms [1,2,19]. Although mushrooms are not among the richest dietary sources of polyphenols compared with berries, tea, cocoa, or coffee, their phenolic profile is relevant because several mushroom-associated phenolics display antioxidant, anti-inflammatory, enzyme-regulating, insulin-sensitizing, and microbiota-modulating activities [87,88].

Oxidative stress is a major driver of type 2 diabetes progression. Chronic hyperglycemia increases reactive oxygen species production, mitochondrial dysfunction, lipid peroxidation, and activation of inflammatory pathways, ultimately impairing insulin signaling and β-cell viability [96]. Mushroom-derived phenolic compounds may counteract these processes by enhancing endogenous antioxidant defenses, reducing ROS accumulation, and modulating redox-sensitive pathways such as NF-κB and Nrf2. These mechanisms are relevant because pancreatic β-cells are particularly vulnerable to oxidative injury due to their relatively low antioxidant capacity [95].

Protocatechuic acid has attracted particular attention because of its ability to modulate insulin signaling and glucose uptake. Experimental evidence shows that protocatechuic acid improved insulin resistance by regulating inter-tissue communication between skeletal muscle, liver, and adipose tissue. In insulin-resistant cellular models, protocatechuic acid increased the expression or activation of key insulin-signaling mediators, including GLUT4, IRS-1, IRS-2, PPAR-γ, phosphorylated AMPK, and phosphorylated Akt, while improving glucose uptake in muscle, liver, and adipocyte systems [87]. These findings are important because GLUT4 translocation and Akt activation are central to peripheral glucose disposal, whereas AMPK activation improves metabolic flexibility and suppresses lipogenic and inflammatory stress [87,94].

Caffeic acid may also be relevant to type 2 diabetes due to its combined antihyperglycemic, anti-inflammatory, antioxidant, lipid-modulating, and microbiota-regulating properties [88,90]. In diet-induced obese mice, caffeic acid reduced adiposity, improved lipid metabolism, increased energy expenditure, and restored gut microbiota composition [78]. These effects are relevant to diabetes because obesity-associated dysbiosis, adipose tissue inflammation, and ectopic lipid accumulation are major contributors to insulin resistance [85,93]. The enrichment of butyrate-producing bacteria observed after caffeic acid administration further suggests that microbiota-derived metabolites may contribute to improved metabolic regulation [88].

Ferulic acid and chlorogenic acid also have mechanistic relevance for type 2 diabetes. Ferulic acid has been associated with attenuation of oxidative stress, inflammation, and insulin resistance-related disturbances [89,91]. Chlorogenic acid has shown effects on glucose and lipid metabolism, gut barrier integrity, intestinal microbiota composition, and metabolic endotoxemia in experimental models. However, as noted above, the strongest evidence for chlorogenic acid derives from coffee and other plant foods rather than mushrooms, so its contribution to mushroom-specific effects should be interpreted cautiously [92].

The interaction between mushroom phenolics and the gut microbiota is especially relevant in type 2 diabetes. Diabetic dysbiosis is often characterized by reduced microbial diversity, depletion of SCFA-producing bacteria, impaired epithelial barrier function, increased intestinal permeability, and metabolic endotoxemia [9,93,96]. Polyphenols and phenolic acids can reach the colon and undergo microbial biotransformation into lower-molecular-weight metabolites with enhanced bioavailability [9]. These metabolites may influence glucose homeostasis by reducing LPS-induced inflammation, strengthening intestinal barrier function, modulating bile acid metabolism, and regulating incretin-related pathways [9,92,93].

SCFAs represent one of the most important downstream mediators of microbiota–host metabolic communication. Butyrate and propionate can stimulate enteroendocrine L-cells to secrete GLP-1 and PYY, hormones involved in insulin secretion, appetite control, gastric emptying, and postprandial glucose regulation. Butyrate also supports epithelial barrier integrity, suppresses inflammatory signaling, and may enhance insulin sensitivity through epigenetic and immunometabolic mechanisms [81]. Although direct evidence linking isolated mushroom phenolics to SCFA-mediated glycemic improvements is still scarce, broader evidence on polyphenol–microbiota interactions supports this mechanistic framework [9].

Inflammation is another key target. Chronic activation of NF-κB and increased production of TNF-α, IL-6, and IL-1β contribute to impaired insulin receptor signaling, adipose tissue dysfunction, endothelial injury, and β-cell stress. Phenolic acids such as protocatechuic acid, caffeic acid, and ferulic acid have demonstrated capacity to reduce inflammatory signaling and oxidative stress while improving mitochondrial and metabolic function. In inflamed adipocyte models, protocatechuic acid reduced inflammatory cytokine production and improved mitochondrial respiration, supporting its relevance in insulin-resistant states Despite this strong mechanistic rationale, clinical evidence specifically evaluating mushroom-derived phenolic compounds in type 2 diabetes remains limited. Most human studies have investigated whole mushrooms rather than isolated phenolic fractions, which prevents attribution of effects exclusively to phenolics. Whole mushrooms contain β-glucans, ergothioneine, polysaccharides, sterols, dietary fibers, vitamins, and minerals, which may exert additive or synergistic effects on glucose metabolism and gut microbiota composition [16,17].

A randomized controlled parallel trial in overweight and moderately obese middle-aged and older adults evaluated the incorporation of 84 g/day of *Agaricus bisporus* and *Pleurotus ostreatus* into a Mediterranean-style dietary pattern for 8 weeks. Mushroom consumption improved fasting serum glucose compared with the control Mediterranean-style diet, although most other cardiometabolic risk factors were not significantly modified. This finding suggests that mushroom intake may positively influence glucose regulation, but the effect cannot be attributed specifically to phenolic compounds because phenolic fractions were not isolated or quantified [17].

Systematic reviews of mushroom consumption and cardiometabolic outcomes also report heterogeneous findings. Some trials suggest improvements in fasting glucose, postprandial glucose, insulin resistance markers, lipid profile, or inflammatory biomarkers, whereas others show minimal or no significant metabolic effects. Limitations include small sample sizes, short intervention periods, heterogeneous mushroom species and doses, lack of standardized preparations, insufficient characterization of bioactive compounds, and absence of microbiome or metabolomic endpoints [17].

Therefore, the current evidence suggests that mushroom-derived phenolic compounds possess considerable mechanistic potential for type 2 diabetes prevention and management through modulation of oxidative stress, inflammation, insulin signaling, mitochondrial function, gut microbiota composition, SCFA production, intestinal permeability, and incretin-related pathways. Among the phenolics detected in mushrooms, protocatechuic acid, caffeic acid, ferulic acid, and chlorogenic acid appear particularly relevant due to their documented effects on AMPK activation, Akt signaling, GLUT4 expression, inflammatory pathways, adipocyte metabolism, and microbiota regulation [87,88,89,90,92]. However, robust clinical validation using standardized phenolic-rich mushroom extracts or purified phenolic fractions is still lacking.

Future studies should include well-characterized prediabetic and diabetic populations, longer intervention periods, controlled dietary backgrounds, and standardized mushroom phenolic profiling. Relevant endpoints should include HbA1c, fasting glucose, fasting insulin, HOMA-IR, oral glucose tolerance, postprandial glucose, GLP-1, PYY, inflammatory biomarkers, intestinal permeability markers, fecal SCFAs, circulating phenolic metabolites, and microbiome/metabolome profiling. Such approaches are essential to determine whether mushroom-derived phenolics can be integrated into precision nutrition strategies targeting microbiota-associated metabolic dysfunction.

### 6.3. Intestinal and Cardiometabolic Health

The intestinal barrier is a central interface between diet, microbiota, immunity, and cardiometabolic health. Disruption of epithelial integrity increases intestinal permeability and facilitates the translocation of microbial products such as lipopolysaccharide, which can promote systemic inflammation, endothelial dysfunction, insulin resistance, dyslipidemia, and cardiometabolic disease progression. Mushroom-derived phenolics may help preserve intestinal homeostasis through direct antioxidant effects, modulation of microbial ecology, and generation of microbial metabolites that reinforce epithelial and immune regulation [9].

At the gut level, phenolic compounds and their microbial metabolites may enhance epithelial barrier function by supporting tight-junction proteins such as occludin, claudins, and zonula occludens-1. They may also attenuate inflammatory signaling by suppressing NF-κB and NLRP3 inflammasome activation, thereby reducing the production of TNF-α, IL-6, IL-1β, and other inflammatory mediators. These mechanisms are particularly relevant in cardiometabolic disorders, where intestinal inflammation and barrier dysfunction contribute to systemic metabolic stress [9,93].

Mushroom phenolics may also influence the intestinal ecosystem by acting as selective substrates or inhibitors within the microbial community. Unlike classical prebiotics such as inulin or fructo-oligosaccharides, phenolics are not primarily defined by carbohydrate fermentability. Instead, they participate in bidirectional interactions with the gut microbiota: they shape microbial composition and activity while being transformed into smaller bioactive metabolites. This prebiotic-like activity may favor SCFA-producing bacteria, reduce potentially pro-inflammatory taxa, and contribute to improved microbial metabolic output [9,93].

Cardiometabolic effects may arise through several interconnected mechanisms. First, improved gut barrier function may reduce metabolic endotoxemia and systemic inflammation. Second, SCFAs and phenolic metabolites may regulate hepatic lipid metabolism, cholesterol synthesis, bile acid metabolism, and peripheral insulin sensitivity. Third, antioxidant and anti-inflammatory actions may improve endothelial function and vascular homeostasis. These pathways provide a mechanistic basis for the reductions in triglycerides and high-sensitivity C-reactive protein reported in some human mushroom intervention studies [16,86,93]. From a quantitative perspective, the available clinical evidence remains limited and heterogeneous. The cited systematic reviews did not provide pooled effect estimates or heterogeneity statistics such as I^2^, mainly because of the small number of eligible trials, differences in mushroom species, intervention forms, doses, study durations, populations, and outcome definitions [16,17]. Instead, the evidence is largely based on qualitative synthesis and study-level comparisons. Overall, the most consistent signals relate to reductions in serum or plasma triglycerides and high-sensitivity C-reactive protein, whereas findings for fasting glucose, HbA1c, blood pressure, total cholesterol, LDL-C, HDL-C, cardiovascular disease, stroke, and type 2 diabetes incidence remain inconsistent or insufficient [16,17]. Importantly, these studies evaluated whole mushrooms or mushroom-containing dietary interventions rather than isolated phenolic-rich mushroom extracts. Therefore, the observed effects cannot be attributed specifically to mushroom-derived phenolic compounds, and the absence of controlled human studies using standardized phenolic-rich fractions represents a primary research gap [16,17]. Therefore, mushroom-derived phenolics should be presented as mechanistically promising but clinically undervalidated. Future clinical studies should move beyond whole-mushroom interventions and directly compare whole mushrooms, polysaccharide-rich fractions, and standardized phenolic-rich extracts. Such studies should include phenolic profiling, microbiome sequencing, metabolomics, SCFA quantification, inflammatory biomarkers, intestinal barrier markers, and clinically meaningful cardiometabolic endpoints.

Overall, the strongest rationale for mushroom phenolics in intestinal and cardiometabolic health lies in their capacity to interact with the gut microbiota, generate bioactive metabolites, reduce oxidative stress, support epithelial barrier integrity, modulate inflammatory pathways, and influence glucose–lipid metabolism. Nevertheless, future studies must move beyond whole-mushroom interventions and incorporate targeted phenolic characterization, standardized extracts, metabolomics, microbiome sequencing, and clinically meaningful cardiometabolic endpoints.

## 7. Challenges and Future Perspectives

Despite the growing interest in mushroom-derived phenolic compounds as modulators of gut microbiota and host metabolism, the field remains at an early stage and is characterized by several conceptual, methodological, analytical, and translational limitations. Current evidence strongly supports the biological relevance of mushroom phenolics, particularly regarding their antioxidant, anti-inflammatory, microbiota-modulating, and metabolism-related activities. However, important challenges still prevent the establishment of clear causal relationships between mushroom-derived phenolic compounds, gut microbial modulation, and clinically meaningful health outcomes.

One of the major limitations concerns the chemical characterization of mushroom phenolic compounds. Although numerous studies report the presence of phenolic acids and flavonoid-like compounds in edible and medicinal mushrooms, substantial inconsistencies remain across the literature. Differences in mushroom species, geographical origin, cultivation substrates, developmental stage, environmental conditions, post-harvest processing, extraction methodology, and analytical platforms strongly influence the reported phenolic profiles. In particular, the detection of flavonoids in mushrooms remains controversial, as fungi lack several canonical biosynthetic pathways associated with plant flavonoid production. Consequently, some compounds tentatively identified as flavonoids may result from analytical misannotation, contamination from cultivation substrates, environmental uptake, or limitations associated with untargeted metabolomic workflows. Future studies should therefore prioritize high-resolution and standardized analytical approaches, including UHPLC-QTOF-MS/MS, Orbitrap-based metabolomics, isotopic validation, and structurally confirmed compound identification rather than tentative annotation alone.

Another major challenge involves the low bioavailability and highly dynamic metabolism of phenolic compounds. Most mushroom-derived phenolics are poorly absorbed in the small intestine and undergo extensive microbial transformation in the colon. While this characteristic supports their potential role as microbiota-associated bioactive compounds, it also complicates mechanistic interpretation. The biological effects observed after mushroom consumption may not result from the parent compounds themselves, but rather from secondary microbial metabolites generated during colonic fermentation. Significant gaps remain in identifying the specific microbial consortia and enzymatic mechanisms responsible for the colonic biotransformation of mushroom-derived phenolics, particularly at the species and functional levels, warranting further in vivo and mechanistic investigations The microbial enzymes, bacterial taxa, metabolic intermediates, and phenolic-derived metabolites associated with mushroom matrices are still insufficiently characterized compared with plant-derived phenolic compounds. Future research should therefore integrate targeted metabolomics, stable isotope tracing, metagenomics, metatranscriptomics, and metabolite flux analysis to better elucidate the colonic fate of mushroom phenolics and their interaction with the intestinal ecosystem.

The complexity of the mushroom matrix itself also represents a critical limitation. Mushrooms contain multiple bioactive constituents, including β-glucans, chitin, heteropolysaccharides, ergothioneine, terpenoids, proteins, peptides, sterols, vitamins, minerals, and other antioxidant molecules. Consequently, the biological effects observed in most experimental and clinical studies cannot be attributed exclusively to phenolic compounds. This issue is particularly relevant in microbiota-related research because fungal polysaccharides are already well-established fermentable substrates capable of modulating gut microbial composition and SCFA production. A major limitation is that most mushroom studies use whole mushroom preparations, crude extracts or polysaccharide-rich fractions, making it difficult to isolate the specific contribution of phenolic acids. As a result, microbiota-related outcomes are often attributable to the combined effects of β-glucans, chitin, heteropolysaccharides, phenolic acids and other fungal constituents. Therefore, distinguishing the direct contribution of phenolic compounds from the effects mediated by β-glucans and other non-digestible mushroom constituents remains a major scientific challenge. Future studies should include purified phenolic fractions, chemically standardized extracts, and comparative experimental designs capable of separating the contribution of phenolics from that of polysaccharides and other fungal metabolites. Additionally, several comparative experimental approaches will be necessary to distinguish the specific contribution of mushroom-derived phenolic compounds from that of fungal fibers and other bioactive constituents, including studies evaluating whole mushrooms, polysaccharide-rich fractions, phenolic-enriched fractions and purified phenolic acids in parallel.

Another important limitation concerns the current use of the term “prebiotic-like”. Although mushroom phenolics may modulate microbial ecology and microbial metabolite production, robust evidence demonstrating selective microbial utilization and direct causal health benefits remains limited. Most available evidence originates from in vitro digestion models, batch colonic fermentation systems, cell culture experiments, and animal studies, whereas well-designed human intervention studies are still scarce. Moreover, the microbiota response to phenolic compounds is highly individualized. Interindividual differences in microbiota composition, microbial gene content, dietary habits, age, metabolic status, medication use, and lifestyle may substantially alter phenolic metabolism and host responsiveness. This gives rise to distinct microbial metabotypes, meaning that two individuals consuming the same mushroom-derived phenolic compounds may generate entirely different microbial metabolites and physiological responses. Future precision nutrition approaches should therefore consider host–microbiota variability, responder/non-responder phenotypes, and personalized microbial metabolism when evaluating the therapeutic relevance of mushroom phenolics.

Clinical translation also remains limited by methodological heterogeneity among studies. Human interventions frequently differ regarding mushroom species, preparation form, extraction method, phenolic concentration, dosage, intervention duration, dietary background, and evaluated endpoints. In many cases, studies evaluate whole mushrooms without detailed phytochemical characterization, preventing clear mechanistic interpretation. A second limitation is the scarcity of human intervention studies specifically designed to evaluate mushroom-derived phenolic compounds. Much of the mechanistic evidence comes from general phenolic literature, in vitro fermentation models, animal studies or studies using plant-derived polyphenols. Therefore, direct extrapolation to mushroom phenolic acids should be made cautiously. Furthermore, few clinical studies simultaneously assess microbiota composition, microbial metabolites, circulating phenolic metabolites, inflammatory biomarkers, intestinal permeability markers, and metabolic outcomes. Future clinical trials should move beyond descriptive observations and adopt integrated multi-omics approaches combining microbiome sequencing, metabolomics, transcriptomics, inflammatory profiling, and gastrointestinal biomarkers. Standardized intervention protocols, adequately powered randomized controlled trials, and long-term dietary studies will be essential to validate the role of mushroom-derived phenolics in intestinal and metabolic health.

The interaction between mushroom phenolics and intestinal barrier function also deserves further investigation. Although several phenolic compounds and their metabolites appear capable of modulating oxidative stress and inflammatory signaling pathways such as NF-κB, MAPK, and Nrf2, direct evidence regarding their effects on tight junction regulation, mucus layer integrity, epithelial permeability, and gut-associated immune responses remains insufficient. Future mechanistic studies should therefore investigate how mushroom phenolic metabolites interact with intestinal epithelial cells, immune cells, and microbial communities under both healthy and dysbiotic conditions.

Another promising but underexplored area concerns the bidirectional relationship between mushroom phenolics and microbial metabolite production. Most current evidence focuses on short-chain fatty acids, particularly acetate, propionate, and butyrate. However, gut microbial metabolism generates a much broader repertoire of bioactive compounds, including indoles, bile acid derivatives, phenylpropionic acids, phenylacetic acids, urolithins, neurotransmitter-related metabolites, and other signaling molecules involved in gut–liver, gut–brain, and gut–immune communication. Understanding how mushroom-derived phenolics influence these metabolic networks may open new perspectives for their application in metabolic, inflammatory, neurodegenerative, and gastrointestinal disorders.

From an industrial and functional food perspective, mushroom-derived phenolics also represent a promising area for innovation. The development of phenolic-enriched mushroom extracts, fermented mushroom-based products, encapsulated delivery systems, synbiotic formulations, and precision-designed functional foods may improve phenolic stability, intestinal delivery, and microbiota-targeted activity. Additionally, advances in biotechnology, controlled cultivation systems, elicitation strategies, pulsed electric field extraction, enzyme-assisted extraction, and fungal fermentation may allow optimization of phenolic yield and bioactivity. However, standardization, safety assessment, bioaccessibility validation, and regulatory approval will be necessary before these approaches can be translated into nutraceutical or clinical applications.

Overall, future research should move from descriptive characterization toward mechanistic and translational validation. The integration of fungal chemistry, microbiology, gastrointestinal physiology, metabolomics, and clinical nutrition will be fundamental to clarify whether mushroom-derived phenolic compounds can truly be considered microbiota-targeted functional compounds with clinically relevant prebiotic-like activity. Although current evidence remains preliminary, the convergence of microbiome science, fungal biotechnology, and precision nutrition strongly suggests that mushroom phenolics may become an important component of future dietary strategies aimed at improving intestinal and metabolic health.

Future research should adopt more actionable and standardized methodological designs. At the preclinical level, gnotobiotic and human microbiota-associated animal models could help determine whether specific microbial consortia are required for the biotransformation of mushroom-derived phenolics and for the generation of bioactive metabolites. These models would also allow causal testing of microbiota-dependent mechanisms under controlled microbial and dietary conditions. In parallel, intervention studies should use standardized phenolic-enriched mushroom extracts with clearly defined chromatographic fingerprints, preferably based on HPLC-DAD, LC-MS/MS or UHPLC-QTOF-MS/MS profiling, including quantification of major phenolic acids and assessment of batch-to-batch reproducibility. Comparative designs evaluating whole mushrooms, polysaccharide-rich fractions, phenolic-enriched fractions and purified phenolic acids in parallel would be particularly useful to separate the effects of phenolics from those of β-glucans, chitin and other fungal constituents. Finally, multi-omics integration combining shotgun metagenomics, metatranscriptomics, targeted and untargeted metabolomics, SCFA profiling, inflammatory markers and intestinal barrier biomarkers should be prioritized to link mushroom phenolic intake with microbial functions, metabolite production and host physiological responses.

## 8. Conclusions

Mushroom-derived phenolic compounds represent an emerging group of bioactive molecules with potential relevance within the microbiota–metabolite–host axis. Current evidence indicates that edible and medicinal mushrooms contain a diverse phenolic profile, mainly composed of low-molecular-weight phenolic acids such as gallic, protocatechuic, caffeic, *p*-coumaric, ferulic, vanillic, syringic, cinnamic and chlorogenic acids. However, their occurrence and concentration vary substantially according to mushroom species, strain, anatomical part, cultivation conditions, geographical origin, processing and extraction methodology. This variability highlights the need for standardized analytical approaches when evaluating the biological relevance of mushroom phenolics.

Due to their limited absorption in the upper gastrointestinal tract, a fraction of mushroom-derived phenolic compounds may reach the colon, where they become available for microbial biotransformation. Through the action of gut microbial enzymes, these compounds can be converted into smaller phenolic metabolites with potentially greater bioavailability and distinct biological activities. In this context, mushroom phenolics may contribute to the modulation of gut microbial ecology, influence microbial metabolic activity, exert selective antimicrobial pressure, and indirectly support short-chain fatty acid-related responses. These effects may be relevant to intestinal barrier function, oxidative stress regulation, inflammatory signaling and metabolic homeostasis. However, their clinical significance remains to be confirmed, particularly because most available evidence derives from in vitro, preclinical or whole-mushroom intervention studies rather than controlled human trials using isolated or standardized phenolic-rich mushroom extracts.

Nevertheless, the classification of mushroom-derived phenolic compounds as true prebiotics remains premature. Unlike classical prebiotics, such as inulin, fructooligosaccharides, galactooligosaccharides and resistant starch, phenolic compounds are not primary fermentable carbohydrate substrates and their effects appear to be highly dependent on chemical structure, dose, food matrix, baseline microbiota composition and host metabolic status. Therefore, the term “prebiotic-like” more accurately reflects their current status as ecological modulators of microbial composition, microbial metabolism and host–microbe signaling.

Importantly, most available evidence derives from whole-mushroom preparations, crude extracts, in vitro digestion and fermentation models, cell culture studies and preclinical experiments. As mushrooms contain β-glucans, chitin, heteropolysaccharides, proteins, ergothioneine, sterols and other bioactive constituents, the specific contribution of phenolic compounds remains difficult to distinguish from the effects of the broader fungal matrix. Human evidence remains limited and is mainly based on whole-mushroom interventions, which prevents direct attribution of intestinal or cardiometabolic benefits to phenolic compounds alone.

Future research should therefore move beyond descriptive phenolic profiling and whole-mushroom interventions toward mechanistic and translational validation. Studies using purified phenolic compounds, phenolic-enriched fractions, standardized mushroom extracts and comparative matrix-based designs are needed to clarify the specific role of mushroom phenolics in gut microbiota modulation. The integration of metabolomics, microbiome sequencing, metagenomics, intestinal barrier markers, inflammatory biomarkers, circulating phenolic metabolites and clinically relevant metabolic endpoints will be essential. Well-designed human trials are particularly required to determine whether mushroom-derived phenolic compounds can be translated into functional foods, nutraceuticals or precision nutrition strategies targeting intestinal and metabolic health.

## Data Availability

All data supporting the findings of this study are available within the paper.

## Supplementary Materials

The following supporting information can be downloaded at https://www.mdpi.com/article/10.3390/ph19071014/s1: Table S1: Phenolic compounds reported in edible mushrooms using HPLC based analytical techniques.

## List of Abbreviations

DW	dry weight
FOSs	fructooligosaccharides
GOSs	galactooligosaccharides
HPLC-DAD-ESI/MS	high-performance liquid chromatography with diode array detection coupled to electrospray ionization mass spectrometry
HPLC-DAD	liquid chromatography with diode-array detection
HPLC	liquid chromatography
ISAPP	international scientific association for probiotics and prebiotics
LC-ESI-QTOF-MS/MS	liquid chromatography coupled to electrospray ionization quadrupole time-of-flight tandem mass spectrometry
LC-MS/MS	liquid chromatography coupled to tandem mass spectrometry
PEFs	pulsed electric fields
SCFA	short-chain fatty acid
UHPLC-QTOF-MS/MS	ultra-high-performance liquid chromatography coupled to quadrupole time-of-flight tandem mass spectrometry
