# Supplementary material for: Mushroom-Derived Phenolic Compounds as Emerging Prebiotic-like Modulators of Gut Microbiota, Intestinal Health, and Metabolism
Source: Pharmaceuticals (Basel). 2026 Jun 30;19(7):1014. doi: 10.3390/ph19071014 (PMC13415312; doi:10.3390/ph19071014)
Supplement: Supplementary file 1 [file pharmaceuticals-19-01014-s001.zip › pharmaceuticals-4347834-supplementary.pdf]

Table S1. Phenolic compounds reported in edible mushrooms using HPLC-based analytical techniques.

Compounds	Mushroom species	Cultivation	Mushroom part	Locatic	Type of extract	Concentration	HPLC-based technique	Ref.
3,5-dicaffeoylquinic acid	*Agaricus bisporus*	Cultivated	Fruiting body	Spain	Aqueous extract (conventional)	1.82 ± 0.20 μg/g DW	Triple TOF-LC-MS/MS	[8]
	Agaricus bisporus	Cultivated	Fruiting body	Spain	Aqueous extract (PEF-assisted)	2.37 ± 0.18 μg/g DW	Triple TOF-LC-MS/MS	
4-hydroxybenzaldehyde	*Agaricus bisporus* (white)	Cultivated	Stems	Poland	Ethanolic extract (80% *v/v*)	0.81 ± 0.03 μg/g DW	LC-MS/MS	[7]
	Agaricus bisporus (white)	Cultivated	Caps	Poland	Ethanolic extract (80% *v/v*)	0.30 ± 0.08 μg/g DW	LC-MS/MS	
	Agaricus bisporus (white)	Cultivated	Whole	Poland	Ethanolic extract (80% *v/v*)	0.41 ± 0.05 μg/g DW	LC-MS/MS	
	Agaricus bisporus (brown)	Cultivated	Stems	Poland	Ethanolic extract (80% *v/v*)	0.68 ± 0.03 μg/g DW	LC-MS/MS	
	Agaricus bisporus (brown)	Cultivated	Caps	Poland	Ethanolic extract (80% *v/v*)	0.51 ± 0.01 μg/g DW	LC-MS/MS	
	Agaricus bisporus (brown)	Cultivated	Whole	Poland	Ethanolic extract (80% *v/v*)	0.55 ± 0.01 μg/g DW	LC-MS/MS	
	Lentinula edodes	Cultivated	Stems	Poland	Ethanolic extract (80% *v/v*)	0.30 ± 0.01 μg/g DW	LC-MS/MS	
	Lentinula edodes	Cultivated	Caps	Poland	Ethanolic extract (80% *v/v*)	0.29 ± 0.02 μg/g DW	LC-MS/MS	
	Lentinula edodes	Cultivated	Whole	Poland	Ethanolic extract (80% *v/v*)	0.29 ± 0.02 μg/g DW	LC-MS/MS	
	Pholiota nameko	Cultivated	Stems	Poland	Ethanolic extract (80% *v/v*)	0.52 ± 0.02 μg/g DW	LC-MS/MS	
	Pholiota nameko	Cultivated	Caps	Poland	Ethanolic extract (80% *v/v*)	1.12 ± 0.05 μg/g DW	LC-MS/MS	
	Pholiota nameko	Cultivated	Whole	Poland	Ethanolic extract (80% *v/v*)	0.98 ± 0.04 μg/g DW	LC-MS/MS	
	Pleurotus eryngii	Cultivated	Stems	Poland	Ethanolic extract (80% *v/v*)	0.60 ± 0.04 μg/g DW	LC-MS/MS	
	Pleurotus eryngii	Cultivated	Caps	Poland	Ethanolic extract (80% *v/v*)	0.71 ± 0.01 μg/g DW	LC-MS/MS	
	Pleurotus eryngii	Cultivated	Whole	Poland	Ethanolic extract (80% *v/v*)	0.68 ± 0.01 μg/g DW	LC-MS/MS	
	Pleurotusostreatus	Cultivated	Stems	Poland	Ethanolic extract (80% *v/v*)	0.60 ± 0.01 μg/g DW	LC-MS/MS	
	Pleurotus ostreatus	Cultivated	Caps	Poland	Ethanolic extract (80% *v/v*)	0.84 ± 0.03 μg/g DW	LC-MS/MS	
	Pleurotus ostreatus	Cultivated	Whole	Poland	Ethanolic extract (80% *v/v*)	0.78 ± 0.02 μg/g DW	LC-MS/MS	
Caffeic acid	*Agaricus bisporus* (white)	Cultivated	Caps	Poland	Ethanolic extract (80% *v/v*)	0.02 ± 0.00 μg/g DW	LC-MS/MS	[7]
	Agaricus bisporus (white)	Cultivated	Whole	Poland	Ethanolic extract (80% *v/v*)	0.01 ± 0.00 μg/g DW	LC-MS/MS	
	Agaricus bisporus (brown)	Cultivated	Stems	Poland	Ethanolic extract (80% *v/v*)	0.04 ± 0.02 μg/g DW	LC-MS/MS	
	Agaricus bisporus (brown)	Cultivated	Caps	Poland	Ethanolic extract (80% *v/v*)	0.35 ± 0.01 μg/g DW	LC-MS/MS	
	Agaricus bisporus (brown)	Cultivated	Whole	Poland	Ethanolic extract (80% *v/v*)	0.28 ± 0.00 μg/g DW	LC-MS/MS	
	Lentinula edodes	Cultivated	Stems	Poland	Ethanolic extract (80% *v/v*)	0.23 ± 0.05 μg/g DW	LC-MS/MS	
	Lentinula edodes	Cultivated	Caps	Poland	Ethanolic extract (80% *v/v*)	0.47 ± 0.12 μg/g DW	LC-MS/MS	
	Lentinula edodes	Cultivated	Whole	Poland	Ethanolic extract (80% *v/v*)	0.41 ± 0.11 μg/g DW	LC-MS/MS	
	Pholiota nameko	Cultivated	Stems	Poland	Ethanolic extract (80% *v/v*)	0.18 ± 0.01 μg/g DW	LC-MS/MS	
	Pholiota nameko	Cultivated	Caps	Poland	Ethanolic extract (80% *v/v*)	0.37 ± 0.02 μg/g DW	LC-MS/MS	

	Pholiota nameko	Cultivated	Whole	Poland	Ethanolic extract (80% *v/v*)	0.33 ± 0.01 μg/g DW	LC-MS/MS	
	Pleurotus ostreatus	Cultivated	Stems	Poland	Ethanolic extract (80% *v/v*)	0.24 ± 0.01 μg/g DW	LC-MS/MS	
	Pleurotus ostreatus	Cultivated	Caps	Poland	Ethanolic extract (80% *v/v*)	0.09 ± 0.01 μg/g DW	LC-MS/MS	
	Pleurotus ostreatus	Cultivated	Whole	Poland	Ethanolic extract (80% *v/v*)	0.12 ± 0.01 μg/g DW	LC-MS/MS	
Cinnamic acid	*Armillaria mellea*	Wild	Fruiting body	Morocco and Portugal	Methanolic extract (100% methanol)	100.60–155.20 μg/g DW	LC-MS	[20]
	Macrolepiota procera	Wild	Fruiting body	Morocco and Portugal	Methanolic extract (100% methanol)	81.93–90.60 μg/g DW	LC-MS	
	Agaricus bisporus	Cultivated	Fruiting body	Spain	Aqueous extract (conventional)	14.23 ± 1.26 μg/g DW	Triple TOF-LC-MS/MS	[8]
	Agaricus bisporus	Cultivated	Fruiting body	Spain	Aqueous extract (PEF-assisted)	20.43 ± 1.03 μg/g DW	Triple TOF-LC-MS/MS	
	Lentinula edodes	Cultivated	Fruiting body	Spain	Aqueous extract (conventional)	3.06 ± 0.23 μg/g DW	Triple TOF-LC-MS/MS	
	Lentinula edodes	Cultivated	Fruiting body	Spain	Aqueous extract (PEF-assisted)	8.59 ± 0.77 μg/g DW	Triple TOF-LC-MS/MS	
Ellagic acid	*Pleurotus ostreatus*	Cultivated	Fruiting body	Spain	Aqueous extract (conventional)	Around 2.72 μg/g DW	Triple TOF-LC-MS/MS	[8]
	Pleurotus ostreatus	Cultivated	Fruiting body	Spain	Aqueous extract (PEF-assisted)	Around 2.65 μg/g DW	Triple TOF-LC-MS/MS	
Ferulic acid	*Agaricus bisporus* (white)	Cultivated	Stems	Poland	Ethanolic extract (80% *v/v*)	0.06 ± 0.00 μg/g DW	LC-MS/MS	[7]
	Agaricus bisporus (white)	Cultivated	Caps	Poland	Ethanolic extract (80% *v/v*)	0.06 ± 0.00 μg/g DW	LC-MS/MS	
	Agaricus bisporus (white)	Cultivated	Whole	Poland	Ethanolic extract (80% *v/v*)	0.06 ± 0.00 μg/g DW	LC-MS/MS	
	Agaricus bisporus (brown)	Cultivated	Stems	Poland	Ethanolic extract (80% *v/v*)	0.06 ± 0.01 μg/g DW	LC-MS/MS	
	Agaricus bisporus (brown)	Cultivated	Caps	Poland	Ethanolic extract (80% *v/v*)	0.06 ± 0.00 μg/g DW	LC-MS/MS	
	Agaricus bisporus (brown)	Cultivated	Whole	Poland	Ethanolic extract (80% *v/v*)	0.06 ± 0.00 μg/g DW	LC-MS/MS	
	Pholiota nameko	Cultivated	Stems	Poland	Ethanolic extract (80% *v/v*)	0.07 ± 0.00 μg/g DW	LC-MS/MS	
	Pholiota nameko	Cultivated	Whole	Poland	Ethanolic extract (80% *v/v*)	0.02 ± 0.00 μg/g DW	LC-MS/MS	
	Armillaria mellea	Wild	Fruiting body	Morocco and Portugal	Methanolic extract (100% methanol)	3.33–18.52 μg/g DW	LC-MS	[20]
	Macrolepiota procera	Wild	Fruiting body	Morocco and Portugal	Methanolic extract (100% methanol)	5.11–6.36 μg/g DW	LC-MS	
Gallic acid	*Armillaria mellea*	Wild	Fruiting body	Morocco and Portugal	Methanolic extract (100% methanol)	32.24 ± 0.45 μg/g DW	LC-MS	[20]
	Macrolepiota procera	Wild	Fruiting body	Morocco and Portugal	Methanolic extract (100% methanol)	11.58–16.41 μg/g DW	LC-MS	

	Armillaria mellea	Wild	Fruiting body	Morocco and Portugal	Methanolic extract (100% methanol)	1.72–6.56 µg/g DW	LC-MS	[20]
	Macrolepiota procera	Wild	Fruiting body	Morocco and Portugal	Methanolic extract (100% methanol)	2.04–3.07 µg/g DW	LC-MS	
p-Coumaric acid	*Agaricus bisporus* (white)	Cultivated	Stems	Poland	Ethanolic extract (80% *v*/*v*)	0.03 ± 0.01 µg/g DW	LC-MS/MS	[7]
	Agaricus bisporus (white)	Cultivated	Caps	Poland	Ethanolic extract (80% *v*/*v*)	0.08 ± 0.01 µg/g DW	LC-MS/MS	
	Agaricus bisporus (white)	Cultivated	Whole	Poland	Ethanolic extract (80% *v*/*v*)	0.07 ± 0.01 µg/g DW	LC-MS/MS	
	Agaricus bisporus (brown)	Cultivated	Stems	Poland	Ethanolic extract (80% *v*/*v*)	0.31 ± 0.02 µg/g DW	LC-MS/MS	
	Agaricus bisporus (brown)	Cultivated	Caps	Poland	Ethanolic extract (80% *v*/*v*)	0.62 ± 0.07 µg/g DW	LC-MS/MS	
	Agaricus bisporus (brown)	Cultivated	Whole	Poland	Ethanolic extract (80% *v*/*v*)	0.55 ± 0.06 µg/g DW	LC-MS/MS	
	Lentinula edodes	Cultivated	Stems	Poland	Ethanolic extract (80% *v*/*v*)	0.02 ± 0.01 µg/g DW	LC-MS/MS	
	Lentinula edodes	Cultivated	Whole	Poland	Ethanolic extract (80% *v*/*v*)	<0.01 µg/g DW	LC-MS/MS	
	Pholiota nameko	Cultivated	Stems	Poland	Ethanolic extract (80% *v*/*v*)	0.39 ± 0.04 µg/g DW	LC-MS/MS	
	Pholiota nameko	Cultivated	Caps	Poland	Ethanolic extract (80% *v*/*v*)	3.79 ± 0.34 µg/g DW	LC-MS/MS	
	Pholiota nameko	Cultivated	Whole	Poland	Ethanolic extract (80% *v*/*v*)	3.00 ± 0.27 µg/g DW	LC-MS/MS	
	Pleurotus eryngii	Cultivated	Stems	Poland	Ethanolic extract (80% *v*/*v*)	0.05 ± 0.01 µg/g DW	LC-MS/MS	
	Pleurotus eryngii	Cultivated	Caps	Poland	Ethanolic extract (80% *v*/*v*)	0.23 ± 0.01 µg/g DW	LC-MS/MS	
	Pleurotus eryngii	Cultivated	Whole	Poland	Ethanolic extract (80% *v*/*v*)	0.19 ± 0.01 µg/g DW	LC-MS/MS	
	Pleurotus ostreatus	Cultivated	Stems	Poland	Ethanolic extract (80% *v*/*v*)	0.06 ± 0.01 µg/g DW	LC-MS/MS	
	Pleurotus ostreatus	Cultivated	Caps	Poland	Ethanolic extract (80% *v*/*v*)	0.07 ± 0.01 µg/g DW	LC-MS/MS	
	Pleurotus ostreatus	Cultivated	Whole	Poland	Ethanolic extract (80% *v*/*v*)	0.07 ± 0.01 µg/g DW	LC-MS/MS	
p-Hydroxybenzoic acid	*Armillaria mellea*	Wild	Fruiting body	Morocco and Portugal	Methanolic extract (100% methanol)	13.00–43.85 µg/g DW	LC-MS	[20]
	Macrolepiota procera	Wild	Fruiting body	Morocco and Portugal	Methanolic extract (100% methanol)	9.99–16.76 µg/g DW	LC-MS	
Paraben	*Armillaria mellea*	Wild	Fruiting body	Poland	Methanolic extract (100% methanol)	17.40–48.12 µg/g DW	LC-MS	[20]
	Macrolepiota procera	Wild	Fruiting body	Poland	Methanolic extract (100% methanol)	15.30–17.87 µg/g DW	LC-MS	
Protocatechuic acid	*Armillaria mellea*	Wild	Fruiting body	Poland	Methanolic extract (100% methanol)	43.90–48.34 µg/g DW	LC-MS	
	Macrolepiota procera	Wild	Fruiting body	Poland	Methanolic extract (100% methanol)	93.52–125.50 µg/g DW	LC-MS	
	Agaricus bisporus (white)	Cultivated	Stems	Poland	Ethanolic extract (80% *v*/*v*)	0.05 ± 0.02 µg/g DW	LC-MS/MS	[7]
	Agaricus bisporus (white)	Cultivated	Caps	Poland	Ethanolic extract (80% *v*/*v*)	0.10 ± 0.04 µg/g DW	LC-MS/MS	

	Agaricus bisporus (white)	Cultivated	Whole	Poland	Ethanolic extract (80% *v/v*)	0.09 ± 0.03 µg/g DW	LC-MS/MS	
	Agaricus bisporus (brown)	Cultivated	Stems	Poland	Ethanolic extract (80% *v/v*)	0.15 ± 0.02 µg/g DW	LC-MS/MS	
	Agaricus bisporus (brown)	Cultivated	Caps	Poland	Ethanolic extract (80% *v/v*)	0.09 ± 0.01 µg/g DW	LC-MS/MS	
	Agaricus bisporus (brown)	Cultivated	Whole	Poland	Ethanolic extract (80% *v/v*)	0.10 ± 0.01 µg/g DW	LC-MS/MS	
	Pholiota nameko	Cultivated	Stems	Poland	Ethanolic extract (80% *v/v*)	0.59 ± 0.02 µg/g DW	LC-MS/MS	
	Pholiota nameko	Cultivated	Caps	Poland	Ethanolic extract (80% *v/v*)	0.93 ± 0.03 µg/g DW	LC-MS/MS	
	Pholiota nameko	Cultivated	Whole	Poland	Ethanolic extract (80% *v/v*)	0.85 ± 0.03 µg/g DW	LC-MS/MS	
	Pleurotus eryngii	Cultivated	Stems	Poland	Ethanolic extract (80% *v/v*)	1.71 ± 0.05 µg/g DW	LC-MS/MS	
	Pleurotus eryngii	Cultivated	Caps	Poland	Ethanolic extract (80% *v/v*)	3.97 ± 0.09 µg/g DW	LC-MS/MS	
	Pleurotus eryngii	Cultivated	Whole	Poland	Ethanolic extract (80% *v/v*)	3.45 ± 0.06 µg/g DW	LC-MS/MS	
	Pleurotus ostreatus	Cultivated	Stems	Poland	Ethanolic extract (80% *v/v*)	0.95 ± 0.03 µg/g DW	LC-MS/MS	
	Pleurotus ostreatus	Cultivated	Caps	Poland	Ethanolic extract (80% *v/v*)	2.31 ± 0.11 µg/g DW	LC-MS/MS	
	Pleurotus ostreatus	Cultivated	Whole	Poland	Ethanolic extract (80% *v/v*)	1.99 ± 0.08 µg/g DW	LC-MS/MS	
Syringic acid	*Armillaria mellea*	Wild	Fruiting body	Morocco and Portugal	Methanolic extract (100% methanol)	7.80 ± 0.33 µg/g DW	LC-MS	[20]
	Macrolepiota procera	Wild	Fruiting body	Morocco and Portugal	Methanolic extract (100% methanol)	0.40–0.44 µg/g DW	LC-MS	
	Agaricus bisporus (white)	Cultivated	Stems	Poland	Ethanolic extract (80% *v/v*)	0.26 ± 0.01 µg/g DW	LC-MS/MS	[7]
	Agaricus bisporus (white)	Cultivated	Caps	Poland	Ethanolic extract (80% *v/v*)	0.22 ± 0.02 µg/g DW	LC-MS/MS	
	Agaricus bisporus (white)	Cultivated	Whole	Poland	Ethanolic extract (80% *v/v*)	0.23 ± 0.01 µg/g DW	LC-MS/MS	
	Agaricus bisporus (brown)	Cultivated	Stems	Poland	Ethanolic extract (80% *v/v*)	0.41 ± 0.03 µg/g DW	LC-MS/MS	
	Agaricus bisporus (brown)	Cultivated	Caps	Poland	Ethanolic extract (80% *v/v*)	0.35 ± 0.01 µg/g DW	LC-MS/MS	
	Agaricus bisporus (brown)	Cultivated	Whole	Poland	Ethanolic extract (80% *v/v*)	0.36 ± 0.02 µg/g DW	LC-MS/MS	
	Lentinula edodes	Cultivated	Stems	Poland	Ethanolic extract (80% *v/v*)	2.18 ± 0.07 µg/g DW	LC-MS/MS	
	Lentinula edodes	Cultivated	Caps	Poland	Ethanolic extract (80% *v/v*)	0.68 ± 0.04 µg/g DW	LC-MS/MS	
	Lentinula edodes	Cultivated	Whole	Poland	Ethanolic extract (80% *v/v*)	1.02 ± 0.05 µg/g DW	LC-MS/MS	
	Pholiota nameko	Cultivated	Stems	Poland	Ethanolic extract (80% *v/v*)	1.21 ± 0.03 µg/g DW	LC-MS/MS	
	Pholiota nameko	Cultivated	Caps	Poland	Ethanolic extract (80% *v/v*)	1.63 ± 0.02 µg/g DW	LC-MS/MS	
	Pholiota nameko	Cultivated	Whole	Poland	Ethanolic extract (80% *v/v*)	1.53 ± 0.02 µg/g DW	LC-MS/MS	
	Pleurotus eryngii	Cultivated	Stems	Poland	Ethanolic extract (80% *v/v*)	0.17 ± 0.02 µg/g DW	LC-MS/MS	
	Pleurotus eryngii	Cultivated	Caps	Poland	Ethanolic extract (80% *v/v*)	0.07 ± 0.01 µg/g DW	LC-MS/MS	

	Pleurotus eryngii	Cultivated	Whole	Poland	Ethanolic extract (80% *v/v*)	0.09 ± 0.01 µg/g DW	LC-MS/MS	
	Pleurotus ostreatus	Cultivated	Stems	Poland	Ethanolic extract (80% *v/v*)	0.28 ± 0.00 µg/g DW	LC-MS/MS	
	Pleurotus ostreatus	Cultivated	Caps	Poland	Ethanolic extract (80% *v/v*)	0.05 ± 0.01 µg/g DW	LC-MS/MS	
	Pleurotus ostreatus	Cultivated	Whole	Poland	Ethanolic extract (80% *v/v*)	0.10 ± 0.00 µg/g DW	LC-MS/MS	
Thymol	*Pleurotus ostreatus*	Cultivated	Fruiting body	Spain	Aqueous extract (Conventional)	5.49 ± 0.38 µg/g DW	Triple TOF-LC-MS/MS	[8]
trans-Cinnamic acid	*Agaricus bisporus* (white)	Cultivated	Stems	Poland	Ethanolic extract (80% *v/v*)	0.29 ± 0.02 µg/g DW	LC-MS/MS	[8]
	Agaricus bisporus (white)	Cultivated	Caps	Poland	Ethanolic extract (80% *v/v*)	1.20 ± 0.12 µg/g DW	LC-MS/MS	
	Agaricus bisporus (white)	Cultivated	Whole	Poland	Ethanolic extract (80% *v/v*)	0.99 ± 0.10 µg/g DW	LC-MS/MS	
	Agaricus bisporus (brown)	Cultivated	Stems	Poland	Ethanolic extract (80% *v/v*)	0.63 ± 0.08 µg/g DW	LC-MS/MS	
	Agaricus bisporus (brown)	Cultivated	Caps	Poland	Ethanolic extract (80% *v/v*)	0.62 ± 0.09 µg/g DW	LC-MS/MS	
	Agaricus bisporus (brown)	Cultivated	Whole	Poland	Ethanolic extract (80% *v/v*)	0.62 ± 0.07 µg/g DW	LC-MS/MS	
	Lentinula edodes	Cultivated	Stems	Poland	Ethanolic extract (80% *v/v*)	0.86 ± 0.06 µg/g DW	LC-MS/MS	
	Lentinula edodes	Cultivated	Caps	Poland	Ethanolic extract (80% *v/v*)	0.83 ± 0.04 µg/g DW	LC-MS/MS	
	Lentinula edodes	Cultivated	Whole	Poland	Ethanolic extract (80% *v/v*)	0.84 ± 0.02 µg/g DW	LC-MS/MS	
	Pholiota nameko	Cultivated	Stems	Poland	Ethanolic extract (80% *v/v*)	0.93 ± 0.04 µg/g DW	LC-MS/MS	
	Pholiota nameko	Cultivated	Caps	Poland	Ethanolic extract (80% *v/v*)	3.16 ± 0.07 µg/g DW	LC-MS/MS	
	Pholiota nameko	Cultivated	Whole	Poland	Ethanolic extract (80% *v/v*)	2.64 ± 0.06 µg/g DW	LC-MS/MS	
	Pleurotus eryngii	Cultivated	Stems	Poland	Ethanolic extract (80% *v/v*)	1.07 ± 0.55 µg/g DW	LC-MS/MS	
	Pleurotus eryngii	Cultivated	Caps	Poland	Ethanolic extract (80% *v/v*)	1.17 ± 0.71 µg/g DW	LC-MS/MS	
	Pleurotus eryngii	Cultivated	Whole	Poland	Ethanolic extract (80% *v/v*)	1.15 ± 0.61 µg/g DW	LC-MS/MS	
	Pleurotus ostreatus	Cultivated	Stems	Poland	Ethanolic extract (80% *v/v*)	0.47 ± 0.28 µg/g DW	LC-MS/MS	
	Pleurotus ostreatus	Cultivated	Caps	Poland	Ethanolic extract (80% *v/v*)	0.80 ± 0.05 µg/g DW	LC-MS/MS	
	Pleurotus ostreatus	Cultivated	Whole	Poland	Ethanolic extract (80% *v/v*)	0.73 ± 0.09 µg/g DW	LC-MS/MS	
Vanillic acid	*Armillaria mellea*	Wild	Fruiting body	Morocco and Portugal	Methanolic extract (100% methanol)	38.02–198.40 µg/g DW	LC-MS	[20]
	Macrolepiota procera	Wild	Fruiting body	Morocco and Portugal	Methanolic extract (100% methanol)	8.42–8.61 µg/g DW	LC-MS	
	Lentinula edodes	Cultivated	Fruiting body	Spain	Aqueous extract (conventional)	8.80 ± 0.82 µg/g DW	Triple TOF-LC-MS/MS	[8]
	Lentinula edodes	Cultivated	Fruiting body	Spain	Aqueous extract (PEF-assisted)	3.15 ± 0.29 µg/g DW	Triple TOF-LC-MS/MS	

DW; dry weight; LC-MS, liquid chromatography–mass spectrometry; LC-MS/MS, liquid chromatography–tandem mass spectrometry; PEF; pulsed electric fields.
